# Trimethylsilyl Compounds for the Interfacial Stabilization of Thiophosphate‐Based Solid Electrolytes in All‐Solid‐State Batteries

**DOI:** 10.1002/advs.202303308

**Published:** 2023-10-22

**Authors:** Kanghyeon Kim, Taehun Kim, Gawon Song, Seonghyun Lee, Min Soo Jung, Seongmin Ha, A. Reum Ha, Kyu Tae Lee

**Affiliations:** ^1^ School of Chemical and Biological Engineering Institute of Chemical Processes Seoul National University 1 Gwanak‐ro, Gwanak‐gu Seoul 08826 Republic of Korea; ^2^ Advanced Battery Development Team 1 Hyundai Motor Company 37 Cheoldobangmulgwan‐ro, Uiwang‐Si Gyeonggi‐do 16082 Republic of Korea

**Keywords:** all‐solid‐state batteries, argyrodite, cathode‐electrolyte interface, solid electrolyte additives, sulfide‐based solid electrolyte

## Abstract

Argyrodite‐type Li_6_PS_5_Cl (LPSCl) has attracted much attention as a solid electrolyte for all‐solid‐state batteries (ASSBs) because of its high ionic conductivity and good mechanical flexibility. LPSCl, however, has challenges of translating research into practical applications, such as irreversible electrochemical degradation at the interface between LPSCl and cathode materials. Even for Li‐ion batteries (LIBs), liquid electrolytes have the same issue as electrolyte decomposition due to interfacial instability. Nonetheless, current LIBs are successfully commercialized because functional electrolyte additives give rise to the formation of stable cathode‐electrolyte interphase (CEI) and solid‐electrolyte interphase (SEI) layers, leading to supplementing the interfacial stability between electrolyte and electrode. Herein, inspired by the role of electrolyte additives for LIBs, trimethylsilyl compounds are introduced as solid electrolyte additives for improving the interfacial stability between sulfide‐based solid electrolytes and cathode materials. 2‐(Trimethylsilyl)ethanethiol (TMS‐SH), a solid electrolyte additive, is oxidatively decomposed during charge, forming a stable CEI layer. As a result, the CEI layer derived from TMS‐SH suppresses the interfacial degradation between LPSCl and LiCoO_2_, thereby leading to the excellent electrochemical performance of Li | LPSCl | LiCoO_2_, such as superior cycle life over 2000 cycles (85.0% of capacity retention after 2000 cycles).

## Graphical abstract

Trimethylsilyl compounds, such as 2‐(trimethylsilyl)ethanethiol (TMS‐SH), are introduced as solid electrolyte additives for improving the interfacial stability between sulfide‐based solid electrolytes and cathode materials. The CEI layer derived from TMS‐SH suppresses the interfacial degradation between LPSCl and LiCoO_2_, thereby leading to the excellent electrochemical performance of Li | LPSCl | LiCoO_2_, such as superior cycle life over 2000 cycles (85.0 % of capacity retention after 2000 cycles).

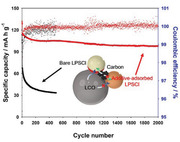

## 1 Introduction

Li‐ion batteries are currently the most widely used type of battery for electric mobility because of their high energy density and long cycle life. However, many people still have concerns about the safety of Li‐ion batteries because they contain flammable organic solvents for electrolytes.^[^ ^1^, ^2^, ^3^, ^4^ ^]^ For this reason, all‐solid‐state batteries (ASSBs) have been considered one of the promising alternatives to replace current Li‐ion batteries because solid electrolytes are considered safer than conventional organic liquid electrolytes. Various types of solid electrolytes have been investigated to improve the electrochemical performance of ASSBs.^[^ ^5^, ^6^, ^7^, ^8^, ^9^, ^10^, ^11^, ^12^, ^13^, ^14^ ^]^ In particular, sulfide‐based thiophosphate solid electrolytes (thio‐SEs), such as argyrodite‐type Li_6_PS_5_Cl (LPSCl), have attracted much attention because of their high ionic conductivity and good mechanical flexibility.^[^ ^15^, ^16^ ^]^ However, thio‐SEs still have challenges of translating research into practical applications, such as poor interfacial stability, although recent efforts showed promise in improving the electrochemical performance of thio‐SEs.^[^ ^17^, ^18^, ^19^ ^]^

Since P─S bond is thermodynamically less stable than P─O bond, thio‐SEs are highly chemically reactive with not only moisture but also oxide‐based cathode materials, leading to the formation of phosphates and oxysulfides on the thio‐SE surface.^[^ ^20^, ^21^, ^22^, ^23^, ^24^, ^25^, ^26^ ^]^ In addition, thio‐SEs are electrochemically oxidized during charge, forming P_2_S_x_ (x > 5) and polysulfide (‐S‐S‐) at the interface between cathode and solid electrolyte. This is attributed to the fact that the electrochemical stability window of thio‐SEs is narrower than the operating voltage range of layered transition metal oxide cathode materials.^[^ ^27^, ^28^ ^]^ The ionic conductivity of these chemically and electrochemically decomposed products on the surface is poorer than that of bulk thio‐SEs, resulting in a significant increase in interfacial resistance. For this reason, the surface coating of oxide cathode materials with protective layers, such as LiNbO_3_, was required to improve the interfacial stability of solid electrolytes.^[^ ^29^, ^30^, ^31^ ^]^ However, the electrochemical decomposition of thio‐SEs was also observed on the carbon additive surface, which is considered another failure mode of thio‐SEs for ASSBs.^[^ ^32^, ^33^, ^34^, ^35^, ^36^ ^]^ In this regard, much effort has been devoted to obtaining chemically and electrochemically stable thio‐SEs. For example, a few surface modifications of thio‐SEs have been introduced to improve i) chemical stability against H_2_O and polar solvents,^[^ ^21^, ^37^, ^38^, ^39^ ^]^ ii) reductive stability with Li‐metal anode,^[^ ^40^, ^41^ ^]^ and iii) chemical and electrochemical stability against oxide cathode materials.^[^ ^42^, ^43^ ^]^ Unfortunately, however, there are currently no completely stable solid electrolytes that meet all requirements for practical ASSBs. Even for Li‐ion batteries containing liquid electrolytes, simple combinations of only salts and solvents are not free from interfacial instability. For this reason, various electrolyte additives have been essentially used to form stable interphases in commercial Li‐ion batteries, such as solid‐electrolyte interphase (SEI) on the anode surface and cathode‐electrolyte interphase (CEI) on the cathode surface.^[^ ^44^ ^]^ This provides insight that the concept of electrolyte additives for thio‐SEs should also be implemented to significantly improve interfacial stability between solid electrolyte and electrode.^[^ ^5^, ^45^ ^]^

Alkylsilanes are known to be promising as electrolyte additives for Li‐ion batteries. For example, trimethylsilyl compounds, such as trimethylsilyl phosphate and trimethylsilyl borate,^[^ ^46^, ^47^ ^]^ are being practically used as electrolyte additives in commercial Li‐ion batteries because they form stable CEI layers, particularly improving the interfacial stability of high voltage cathode materials. In this regard, we introduced trimethylsilyl compounds, including 2‐(trimethylsilyl)ethanethiol, as solid electrolyte additives to form stable CEI layers between argyrodite‐type solid electrolyte Li_6_PS_5_Cl (denoted as LPSCl) and LiCoO_2_ for ASSBs, eventually leading to improving the chemical and electrochemical stability of LPSCl. Liquid electrolyte additives for Li‐ion batteries, which are dissolved in liquid electrolytes, are electrochemically decomposed on the cathode surface, forming stable and uniform CEI layers on the cathode surface. For ASSBs, however, molecular compounds as solid electrolyte additives cannot be soluble in solid electrolytes. For this reason, challenges for solid electrolyte additives are not limited to the formation of stable CEI but also include uniform and thin distribution of solid electrolyte additives between cathode and solid electrolyte. Moreover, in contrast to liquid electrolyte additives for Li‐ion batteries, another role of solid electrolyte additives is to form stable protective layers on the carbon additive surface because solid electrolytes are also electrochemically degraded on the carbon additive surface. For these reasons, solid electrolyte additives were preferably deposited on the LPSCl surface rather than the cathode surface. We also compared various trimethylsilyl compounds, such as 2‐(trimethylsilyl)ethanethiol with a thiol functional group, 2‐(trimethylsilyl)ethanol with a hydroxyl functional group, and bis(trimethylsilyl)methane with no functional groups, as solid electrolyte additives to demonstrate the role of functional groups in the uniform and thin molecular adsorption of additives on the LPSCl surface. 2‐(Trimethylsilyl)ethanethiol remarkably improved the interfacial stability of LPSCl for LiCoO_2_ and carbon additives, resulting in excellent electrochemical performance, such as stable capacity retention over 2000 cycles for Li | LPSCl | LiCoO_2_ at 30 °C. We also examined 1‐dodecanethiol as a solid electrolyte additive to clarify the role of a trimethylsilyl functional group of trimethylsilyl compounds in the formation of stable CEI layers. In addition, 2‐(trimethylsilyl)ethanethiol showed the improved chemical stability of LPSCl in a dry oxygen atmosphere, thus suppressing the degradation of LPSCl in dry air. This suggests that 2‐(trimethylsilyl)ethanethiol is also promising in terms of an industrial cell assembly process.

## 2 Results and Discussion

### 2.1 Synthesis of LPSCl with Solid Electrolyte Additives

**Figure 1:**
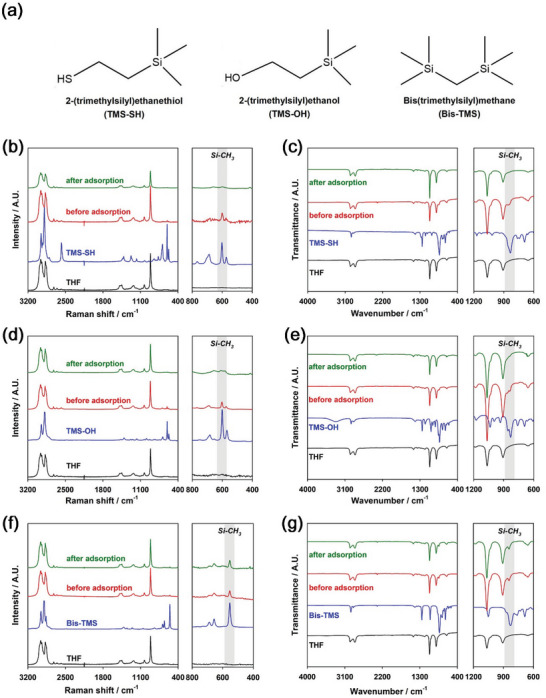
Synthesis of LPSCl powders adsorbed with solid electrolyte additives. a) 2D molecular structures of TMS‐SH, TMS‐OH, and Bis‐TMS. b–g) Raman and IR spectra of bare THF solution, solid electrolyte additives, and solid electrolyte additives‐containing THF solutions before and after adsorption of solid electrolyte additives on the LPSCl surface: b,c) TMS‐SH, d,e) TMS‐OH, and f,g) Bis‐TMS. Black, blue, red, and green spectra represent THF, solid electrolyte additives, and solid electrolyte additives‐containing THF solutions before and after adsorption of solid electrolyte additives on LPSCl, respectively. The fingerprint regions of the Raman (400−800 cm^−1^) and IR (600−1200 cm^−1^) spectra are expanded for clarity.

The electrolyte additives of trimethylsilyl compounds are known to improve the electrochemical performance of high‐voltage cathode materials for Li‐ion batteries.^[^ ^46^, ^47^ ^]^ They contain in common a trimethylsilyl group that leads to the formation of stable CEI layers. In this regard, we examined three trimethylsilyl compounds, such as 2‐(trimethylsilyl)ethanethiol (denoted as TMS‐SH), 2‐(trimethylsilyl)ethanol (denoted as TMS‐OH), and bis(trimethylsilyl)methane (denoted as Bis‐TMS), as solid electrolyte additives. Their molecular structures are displayed in **Figure** **1**a. TMS‐SH and TMS‐OH have thiol and hydroxyl functional groups, respectively, whereas Bis‐TMS has no functional groups except for the common trimethylsilyl group. Those functional groups were intended to provide the role of molecular surface adsorption of trimethylsilyl compounds on the LPSCl surface through hydrogen bonding and chalcogen‐chalcogen interaction.^[^ ^48^ ^]^ LPSCl powders were stirred in 0.1 m trimethylsilyl compounds‐containing tetrahydrofuran (THF) solutions for 3 h, followed by centrifuge to remove excess trimethylsilyl compounds that were not adsorbed on the LPSCl surface. Then, LPSCl powders adsorbed with trimethylsilyl compounds were heated in a vacuum at 40 °C for 12 h to completely remove THF. The adsorption of the solid electrolyte additives on the LPSCl surface was supported by Raman spectroscopy, infrared (IR) spectroscopy, energy dispersive X‐ray spectroscopy (EDS), and X‐ray photoelectron spectroscopy (XPS). Figure 1b–g compares the Raman and IR spectra of trimethylsilyl compounds‐containing THF solutions before and after adsorption of trimethylsilyl compounds on the LPSCl surface. Pristine trimethylsilyl compounds‐containing THF solutions, denoted as before adsorption in Figure 1, show intense characteristic peaks at ca. 600 and 820 cm^−1^, which are assigned to Si‐CH_3_ vibration, in their Raman and IR spectra, respectively.^[^ ^49^, ^50^ ^]^ For TMS‐SH and TMS‐OH, the supernatant solutions retrieved after the centrifuge, denoted as after adsorption in Figure 1, showed significant decreases in intensity of these peaks compared to the pristine solutions. This reveals that the concentrations of TMS‐SH and TMS‐OH in the retrieved solutions decreased compared to the pristine solutions because significant amounts of TMS‐SH and TMS‐OH were adsorbed on the LPSCl surface. In contrast to TMS‐SH and TMS‐OH, Bis‐TMS showed negligible changes in the intensity of characteristic peaks before and after adding LPSCl powders. This indicates that Bis‐TMS was negligibly adsorbed on the LPSCl surface. This contrasting behavior implies that the adsorption of trimethylsilyl compounds on the LPSCl surface was due to hydrogen bonding and chalcogen–chalcogen interaction between the LPSCl surface and the thiol and hydroxyl functional groups of TMS‐SH and TMS‐OH, respectively. The peak position of Si‐CH_3_ in Bis‐TMS was redshifted compared to those of TMS‐SH and TMS‐OH. This was attributed to the fact that the Si‐CH_3_ stretching vibration was affected by the β‐stabilizing effect due to the hyperconjugation of an additional trimethylsilyl group in Bis‐TMS.^[^ ^50^ ^]^ The role of thiol and hydroxyl functional groups in the surface adsorption of trimethylsilyl compounds was also supported by the scanning electron microscopy (SEM)‐EDS mapping images and the corresponding EDS spectra of i) bare LPSCl powders and ii) LPSCl powders retrieved after adsorption with TMS‐SH, TMS‐OH, and Bis‐TMS (Figures S1 and S2, Supporting Information). Si element was clearly and uniformly observed on the LPSCl powders for TMS‐SH and TMS‐OH, whereas a negligible amount of Si element was detected on the LPSCl surface for Bis‐TMS. This implies that TMS‐SH and TMS‐OH were uniformly adsorbed on the LPSCl surface because of hydrogen bonding and chalcogen‐chalcogen interaction.

**Figure 2:**
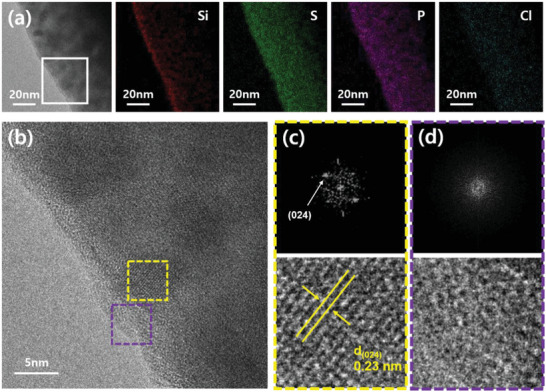
Material characterization of LPSCl powders adsorbed with TMS‐SH. a) TEM and EDS mapping images of LPSCl powders adsorbed with TMS‐SH. Red, green, violet, and blue colors represent silicon, sulfur, phosphorus, and chlorine elements, respectively. b) Magnified TEM image of the selected area (white box) in (a). c,d) FFT patterns and inverse‐FFT images of the selected areas of c) yellow and d) violet boxes in (b), respectively.

**Figure** **2**a presents the transmission electron microscopy (TEM)‐EDS mapping images of LPSCl powders adsorbed with TMS‐SH. The EDS mapping image of Si element reveals that the TMS‐SH was adsorbed on the LPSCl surface. To elucidate the thickness and structure of the coating layer of TMS‐SH, we analyzed the magnified TEM image of the selected area (white box) near the surface in Figure 2a, as shown in Figure 2b. We clearly observed that the amorphous surface layer was adsorbed on the crystalline bulk. The fast Fourier transform (FFT) patterns and their corresponding inverse‐FFT images were obtained for the selected areas in Figure 2b. This supports the finding that the bulk was crystalline (Figure 2c), whereas the surface appeared to be amorphous (Figure 2d). The d‐spacing of the crystalline domain in the bulk was ≈0.23 nm, corresponding to the (024) plane of LPSCl.^[^ ^51^ ^]^ Combining the EDS mapping image and the FFT analysis of the LPSCl powders adsorbed with TMS‐SH, the thickness of the amorphous TMS‐SH coating layer was approximately a few nm. The TEM‐EDS mapping images of LPSCl powders adsorbed with TMS‐OH are also presented in Figure S3 (Supporting Information). The corresponding EDS spectra of LPSCl powders adsorbed with TMS‐SH and TMS‐OH are presented in Figure S4 (Supporting Information). In addition, the amount of TMS‐SH adsorbed on the LPSCl surface was ca. 5 wt.%, which was measured using inductively coupled plasma‐atomic emission spectroscopy (ICP‐AES) analysis (Table S1, Supporting Information).

**Figure 3:**
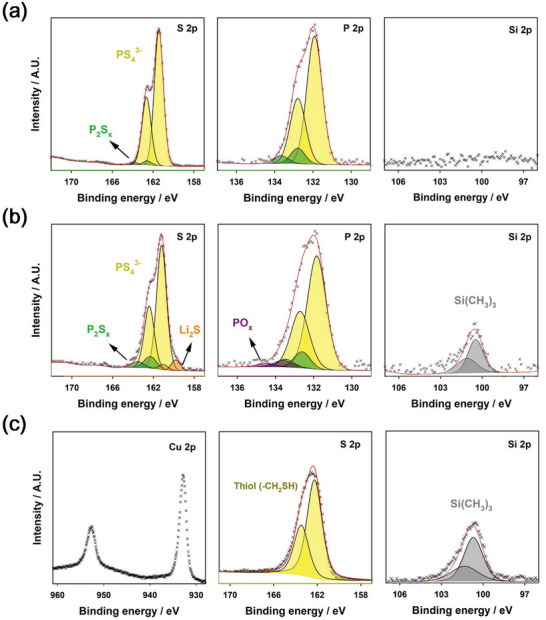
XPS spectra of LPSCl powders adsorbed with TMS‐SH. a‐c) S 2p, P 2p, Si 2p, and Cu 2p XPS spectra of a) bare LPSCl, b) LPSCl adsorbed with TMS‐SH, and c) TMS‐SH adsorbed on the Cu foil surface.

**Figure 4:**
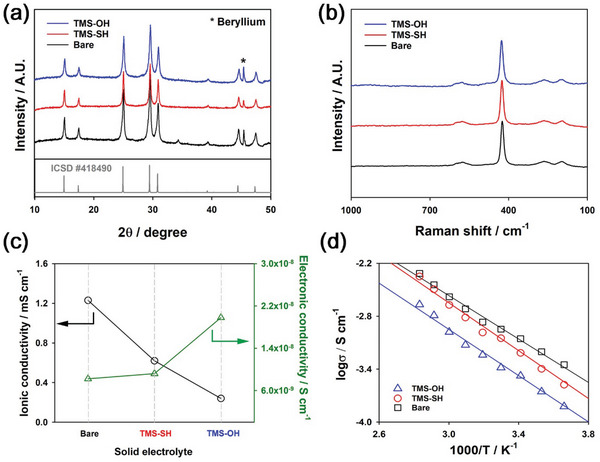
Crystal structure and conductivity of LPSCl adsorbed with trimethylsilyl compounds. a) XRD patterns, b) Raman spectra, c) ionic and electronic conductivities, and d) ionic conductivity at various temperatures for bare LPSCl and LPSCl adsorbed with TMS‐SH and TMS‐OH. Bare, TMS‐OH, and TMS‐SH in the figure legends represent bare LPSCl, LPSCl adsorbed with TMS‐OH, and LPSCl adsorbed with TMS‐SH, respectively. The activation energy was calculated from a slope of the fitted linear regression lines in (d).

The XPS spectra of bare LPSCl, LPSCl adsorbed with TMS‐SH, and TMS‐SH adsorbed on the Cu foil surface, also evidenced the TMS‐SH adsorption on LPSCl surface (**Figure** **3**). The binding energy of S 2p in TMS‐SH, which ranges from 166 to 159 eV, was overlapped with those of in P_2_S_x_ (with x>5) and PS_4_^3−^ building block in LPSCl.^[^ ^22^, ^23^, ^32^ ^]^ However, the Si 2p XPS peak at ca. 101 eV, which corresponds to −Si(CH_3_)_3_, was identically observed only for the LPSCl adsorbed with TMS‐SH and the TMS‐SH adsorbed on the Cu foil surface.^[^ ^52^ ^]^ This reveals that TMS‐SH was effectively adsorbed on the LPSCl surface. We also compared the XRD patterns and Raman spectra of bare LPSCl powders and LPSCl powders adsorbed with TMS‐SH and TMS‐OH, as shown in **Figure** **4**a,b, respectively. Negligible changes were observed before and after the surface adsorption of TMS‐SH and TMS‐OH. This implies that the LPSCl crystal structure was not deformed during the synthesis with THF solution because LPSCl was only exposed to THF for a short time (3 h). In addition, we compared the ionic and electronic conductivities of bare LPSCl powders and LPSCl powders adsorbed with TMS‐SH and TMS‐OH at room temperature, as shown in Figure 4c and Tables S2 and S3 (Supporting Information). The corresponding Nyquist plots and chronoamperometry profiles are presented in Figure S5 (Supporting Information). LPSCl powders adsorbed with TMS‐SH and TMS‐OH showed slightly lower ionic conductivity and higher electronic conductivity than did bare LPSCl. This reveals that the molecular adsorption layers of TMS‐SH and TMS‐OH gave rise to slightly sluggish ionic transport for charge transfer. However, decrease in the ionic conductivity of LPSCl adsorbed with TMS‐SH was insignificant because the TMS‐SH layer was sufficiently thin (Figure 2). The ionic conductivity of LPSCl adsorbed with TMS‐SH (0.62 mS cm^−1^ at room temperature) was also sufficiently high for use as a solid electrolyte. Moreover, Figure 4d shows the ionic conductivity of bare LPSCl and LPSCl adsorbed with TMS‐SH and TMS‐OH at various temperatures. The activation energies of LPSCl adsorbed with TMS‐SH (0.269 eV) and TMS‐OH (0.260 eV) were slightly higher than that of bare LPSCl (0.244 eV). Besides, LPSCl adsorbed with TMS‐OH showed poorer ionic conductivity in all temperature ranges than did LPSCl adsorbed with TMS‐SH. This is attributable to the degradation of the LPSCl surface due to a chemical reaction between LPSCl and the OH group of TMS‐OH, forming oxy‐sulfide LPSCl species, as evidenced by the TOF‐SIMS analysis (Figure S6, Supporting Information). In this regard, TMS‐SH was considered more suitable as a solid electrolyte additive.

### 2.2 Electrochemical Performance

**Figure 5:**
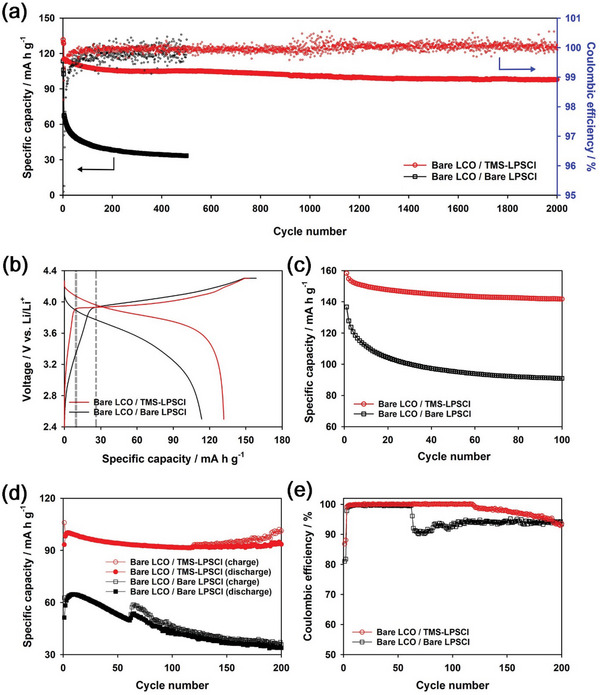
Electrochemical performances of ASSBs. a) Long‐term cycle performance (discharge capacity and Coulombic efficiency) of Li | LPSCl | LiCoO_2_ cells for bare LPSCl and TMS‐LPSCl at 0.11 mA cm^−2^ for charge (CC/CV mode) and 1.1 mA cm^−2^ for discharge (CC mode) in the voltage range of 2.5–4.3 V (vs Li/Li^+^) at 30 °C after precycling. b) Voltage profiles of the initial cycle during precycling at 0.11 mA cm^−2^ and 30 °C. c) Cycle performances (discharge capacity) of bare LPSCl and TMS‐LPSCl at 0.11 mA cm^−2^ for both charge and discharge using a CC/CV mode in the voltage ranges of 2.5–4.5 V (vs Li/Li^+^) at 30°C. d) Charge/discharge capacities and e) the corresponding Coulombic efficiencies of bare LPSCl and TMS‐LPSCl at 0.44 mA cm^−2^ for charge and 1.1 mA cm^−2^ for discharge using a CC mode in the voltage range of 2.5–4.3 V (vs Li/Li^+^) at 30 °C.

We compared the electrochemical performances of bare LiCoO_2_, which was not coated with a protective layer, such as LiNbO_3_, for (i) bare LPSCl and (ii) LPSCl adsorbed with TMS‐SH (denoted as TMS‐LPSCl). ASSBs were fabricated using a bulk‐type cell with the tri‐layers of a composite cathode pellet, a solid electrolyte pellet, and a Li metal foil as anode. The schematic image of the bulk‐type cell is presented in Figure S7 (Supporting Information). The mass loading of LiCoO_2_ was ≈6.8 mg cm^−2^. **Figure** **5**a shows the cycle performances and Coulombic efficiencies of bare LiCoO_2_ for bare LPSCl and TMS‐LPSCl in the voltage range of 2.5−4.3 V (vs Li/Li^+^) at 30 °C. The cells were charged at a current density of 0.11 mA cm^−2^ and discharged at a current density of 1.1 mA cm^−2^ after precycling. Charge was carried out using a constant current/constant voltage (CC/CV) mode in which a cell voltage was held at 4.3 V (vs Li/Li^+^) until current density was decayed to 0.05 mA cm^−2^. The precycling was carried out at 0.11 mA cm^−2^ for 3 cycles at 30 °C. TMS‐LPSCl exhibited excellent cycle performance, such as 85.0% of capacity retention after 2000 cycles, which was superior to bare LPSCl (49.7% of capacity retention after 500 cycles). Note that the sloping voltage profile in the voltage range of 2.5–3.9 V (vs Li/Li^+^) during charge is known to be due to the electrochemical oxidative decomposition of LPSCl.^[^ ^33^, ^53^ ^]^ Bare LPSCl showed a significant amount of charge capacity (ca. 25 mA h g^−1^) in the sloping region for the initial cycle, whereas TMS‐LPSCl showed a negligible amount of charge capacity (ca. 8 mA h g^−1^) in the same region (Figure 5b). This implies that TMS‐SH formed stable passivation layers on the LPSCl surface, suppressing the electrochemical decomposition of LPSCl during the initial charge. As a result, TMS‐LPSCl showed smaller overpotential and higher Coulombic efficiency during cycling than did bare LPSCl (Figure S8, Supporting Information). We also compared the cycle performances and Coulombic efficiencies of bare LiCoO_2_ for bare LPSCl and TMS‐LPSCl in another voltage range of 2.5−4.5 V (vs Li/Li^+^) at the current density of 0.11 mA cm^−2^ for both charge and discharge and at 30 °C using the CC/CV mode (Figure 5c and Figure S9a, Supporting Information, respectively). TMS‐LPSCl showed higher reversible capacity and more stable capacity retention over 100 cycles than did bare LPSCl. This suggests that the interfacial layer derived from TMS‐SH was stable even at high SOC levels, such as 4.5 V (vs Li/Li^+^).

In addition, we examined the electrochemical performances of Li | LPSCl | LiCoO_2_ cells at a high charge current density using a CC mode in the voltage range of 2.5–4.3 V (vs Li/Li^+^) at 30 °C. Figure 5d,e and Figure S10 (Supporting information), show the cycle performances and Coulombic efficiencies for bare LPSCl and TMS‐LPSCl at various charge current densities, such as 0.44 and 0.66 mA cm^−2^. The cells were discharged at a current density of 1.1 mA cm^−2^. The corresponding voltage profiles are displayed in Figure S11 (Supporting Information). TMS‐LPSCl exhibited higher reversible capacity and more stable cycle performance than did bare LPSCl even at both charge current densities of 0.44 and 0.66 mA cm^−2^. However, it is notable that the charge capacities suddenly started to increase after specific cycle numbers for both bare LPSCl and TMS‐LPSCl, whereas the discharge capacities were not changed significantly. As a result, abrupt decreases in Coulombic efficiency were observed after specific cycle numbers (Figure 5e; Figure S10b, Supporting Information). This is attributable to the micro‐short circuit between cathode and Li metal anode due to the formation of Li dendrites at a high charge current density. However, it is remarkable that the micro‐short circuit of TMS‐LPSCl occurred after a longer cycling compared to bare LPSCl at both charge current densities of 0.44 and 0.66 mA cm^−2^. This implies that TMS‐LPSCl suppressed the growth of Li dendrites during cycling compared to bare LPSCl. Note that TMS‐LPSCl was used only for the composite cathode pellet and not for the solid electrolyte pellet. This implies that the suppressed Li dendrite formation of TMS‐LPSCl was not attributable to the SEI layer between LPSCl and Li metal, because only bare LPSCl was used for the solid electrolyte pellet regardless of using bare LPSCl and TMS‐LPSCl for the composite cathodes. Therefore, this reveals that the Li dendrite formation was correlated with the interfacial stability of the composite cathode.

**Figure 6:**
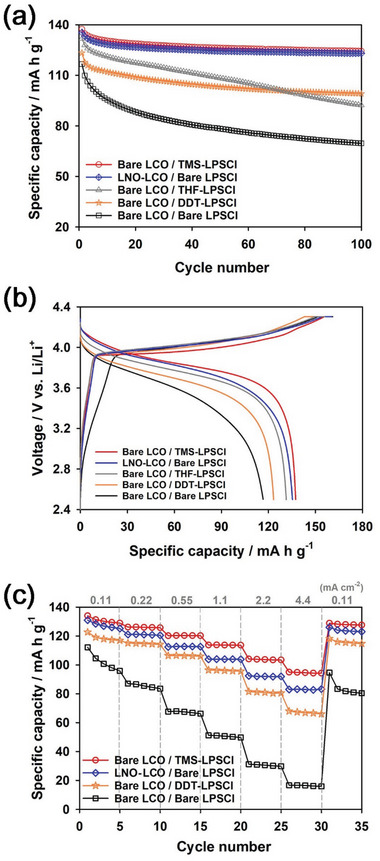
Electrochemical performances of Li | LPSCl | LiCoO_2_ cells for various composite electrodes. a) Cycle performances (discharge capacity) of various composite electrodes at 0.11 mA cm^−2^ for both charge and discharge using a CC/CV mode in the voltage range of 2.5–4.3 V (vs Li/Li^+^) at 30°C. b) Voltage profiles of various composite electrodes for the initial cycle in (a). c) Rate performances (discharge capacity) of various composite electrodes at various discharge current densities in the voltage ranges of 2.5–4.3 V (vs Li/Li^+^) at 30 °C. LiCoO_2_ and LiNbO_3_ were denoted as LCO and LNO, respectively.

To clarify the role of the trimethylsilyl group in the improved electrochemical performance of TMS‐SH, we compared TMS‐SH and 1‐dodecanethiol (denoted as DDT‐SH) as solid electrolyte additives. DDT‐SH contains a thiol group but not a trimethylsilyl group, as shown in Figure S12a (Supporting Information). DDT‐SH was adsorbed on the LPSCl surface using the same procedure as TMS‐SH. The Raman and IR spectra of THF solutions containing DDT‐SH before and after adsorption of DDT‐SH on the LPSCl surface are presented in Figure S12b,c (Supporting Information), respectively. Pristine THF solutions of DDT‐SH show intense characteristic peaks at ca. 2580 cm^−1^ in the Raman spectrum and at ca. 2930 cm^−1^ in the IR spectrum, which are assigned to S‐H and ‐CH_2_‐ stretching vibrations, respectively.^[^ ^54^, ^55^ ^]^ The intensity of these peaks decreased significantly when we measured the supernatant solutions retrieved after the centrifuge compared to the pristine solutions, implying that a significant amount of DDT‐SH was adsorbed on the LPSCl surface. LPSCl adsorbed with 1‐dodecanethiol (denoted as DDT‐LPSCl) showed poorer cycle performance in the voltage range of 2.5 − 4.3 V (vs Li/Li^+^) at a current density of 0.11 mA cm^−2^ and 30 °C using the CC/CV mode than did TMS‐LPSCl, although DDT‐LPSCl improved the cycle performance compared to bare LPSCl (**Figure** **6**a). The corresponding voltage profiles for the initial cycle are presented in Figure 6b. This supports that the trimethylsilyl group played a decisive role in forming a stable passivation layer on the LPSCl surface. This is also supported by the discharge rate performances of TMS‐LPSCl superior to DDT‐LPSCl and bare LPSCl (Figure 6c). The cells were discharged at various current densities, whereas the cells were charged at the same current density of 0.11 mA cm^−2^ using the CC/CV mode in the voltage range of 2.5–4.3 V (vs Li/Li^+^). TMS‐LPSCl delivered a specific capacity of 100 mA h g^−1^ at 4.4 mA cm^−2^, whereas bare LPSCl and DDT‐LPSCl delivered 17 and 67 mA h g^−1^ at 4.4 mA cm^−2^, respectively. This implies that the CEI layer derived from TMS‐SH suppressed the charge transfer resistance at the interface between LiCoO_2_ and LPSCl compared to bare LPSCl.

We also examined the electrochemical performance of LPSCl exposed to THF solvent without additives to demonstrate the role of THF in the electrochemical performance of TMS‐LPSCl. LPSCl powders exposed to THF solvent (denoted as THF‐LPSCl) were obtained using the same procedure as for the synthesis of TMS‐LPSCl except for the addition of TMS‐SH. The S 2p and P 2p XPS spectra of THF‐LPSCl powders are presented in Figure S13 (Supporting Information). The XPS peaks (yellow) at 161.4 and 131.9 eV correspond to the S 2p and P.

2p signals of the PS_4_^3−^ tetrahedra (P−S−Li bonds) in the argyrodite LPSCl structure.^[^ ^23^, ^31^ ^]^ In contrast to bare LPSCl (Figure 3a), we observed more intense signals of P_2_S_x_ (x > 5) (green), Li_2_S (orange), and phosphate species (PO_y_ : violet) for THF‐LPSCl. This reveals that the THF‐LPSCl surface was slightly oxidized during storage of LPSCl powders in THF solvent although its bulk structure was not deformed. TMS‐LPSCl powders also showed the almost same XPS spectra as THF‐LPSCl, implying that TMS‐LPSCl was also slightly oxidized because of THF solvent during the synthesis (Figure 3b). For this reason, the ionic conductivity of THF‐LPSCl (0.68 mS cm^−1^ at room temperature) was lower than that of bare LPSCl (1.23 mS cm^−1^ at room temperature), but slightly higher than that of TMS‐LPSCl (0.62 mS cm^−1^ at room temperature), as shown in Figure S14 and Table S4 (Supporting Information). The cycle performance and the corresponding voltage profile of Li | LPSCl | LiCoO_2_ for THF‐LPSCl are also presented in Figure 6a,b, respectively. THF‐LPSCl showed higher reversible capacity and more stable capacity retention compared to bare LPSCl. This is attributed to the fact that the slight oxidation of the LPSCl surface due to THF exposure formed the passivation layer consisting of P_2_S_x_, Li_2_S, and PO_y_, suppressing the electrochemical decomposition of LPSCl during cycling.^[^ ^42^ ^]^ In addition, LPSCl is known to become softer and more deformable after exposure to THF solvent.^[^ ^56^ ^]^ This suggests that the improved electrochemical performance of THF‐LPSCl is also attributable to the densification of the LPSCl pellet due to THF solvent. However, the electrochemical performance of TMS‐LPSCl was superior to that of THF‐LPSCl, despite the fact that both TMS‐LPSCl and THF‐LPSCl were similarly densified after exposure to THF solvent during the synthesis. The capacity retention of THF‐LPSCl (70.0% after 100 cycles) was poorer than that of TMS‐LPSCl (90.5% after 100 cycles). The overpotential of THF‐LPSCl was also larger than that of TMS‐LPSCl. This reveals that the CEI layer derived from TMS‐SH was more stable than the passivation layer due to the THF exposure, leading to the improved electrochemical performance of TMS‐LPSCl. In addition, we compared the electrochemical performances of bare LiCoO_2_ with TMS‐LPSCl and LiNbO_3_‐coated LiCoO_2_ with bare LPSCl. LiNbO_3_‐coated LiCoO_2_ was synthesized through a conventional sol‐gel method.^[^ ^14^, ^29^ ^]^ The SEM/EDS mapping images and XRD pattern of LiNbO_3_‐coated LiCoO_2_ are presented in Figure S15 (Supporting Information). Although LiNbO_3_‐coated LiCoO_2_ also showed stable capacity retention and high Coulombic efficiency over 100 cycles (Figure 6a; Figure S9b, Supporting Information), the rate performance of bare LiCoO_2_ with TMS‐LPSCl was significantly better than that of LiNbO_3_‐coated LiCoO_2_ with bare LPSCl (ca. 83 mA h g^−1^ at 4.4 mA cm^−2^), as shown in Figure 6c. This implies that the CEI layer derived from TMS‐SH showed smaller charge transfer resistance than did the LiNbO_3_ protective layer. This is also supported by the smaller overpotential and higher Coulombic efficiency of bare LiCoO_2_ with TMS‐LPSCl compared to LiNbO_3_‐coated LiCoO_2_ with bare LPSCl, as shown in their voltage profiles (Figure 6b). We also compared the electrochemical performances of LiCoO_2_ for TMS‐LPSCl with those of LiCoO_2_ reported in recent literatures, as shown in Figure S16 and Table S5 (Supporting Information).

In addition, we optimized the amounts of the TMS‐SH additive adsorption layer in terms of ionic conductivity, rate capability, and cycle performance. TMS‐SH was adsorbed on the LPSCl powder surface using various concentrations of TMS‐SH in THF, such as 0.05, 0.1, and 0.2 m. As shown in Figure S17 and Table S6 (Supporting Information), the ionic conductivity of LPSCl adsorbed with TMS‐SH decreased with increasing the concentration of TMS‐SH in THF. However, since the ionic conductivity of LPSCl adsorbed with TMS‐SH decreased significantly between 0.1 and 0.2 m, 0.1 m was selected as the optimal concentration of TMS‐SH. The amounts of TMS‐SH adsorbed on the LPSCl surface were measured using ICP‐AES analysis (Table S7, Supporting Information). Figure S18a (Supporting Information) shows the voltage profiles of TMS‐LPSCl for various concentrations of TMS‐SH, such as 0.05, 0.1, and 0.2 m, in the voltage range of 2.5–4.3 V (vs Li/Li^+^) at 30 °C. The cells were examined using two current protocols for charge and discharge. The first protocol involved charging and discharging the cells at a 0.1C rate (0.11 mA cm^−2^). The second protocol involved charging at a 0.2C rate (0.22 mA cm^−2^) and discharging at a 0.5C rate (0.54 mA cm^−2^). TMS‐LPSCl delivered the highest reversible capacity when the concentration of TMS‐SH was 0.1 m. Moreover, TMS‐LPSCl showed stable capacity retention at a concentration of 0.1 m TMS‐SH, as shown in Figure S18b (Supporting Information). Therefore, the optimized concentration of TMS‐SH in terms of rate capability and cycle performance was determined to be 0.1 m, which aligns with the optimized ionic conductivity. This reveals that the coverage of TMS‐SH on the LPSCl was not complete at a TMS‐SH concentration of 0.05 m, leading to poor electrochemical performance due to significant degradation of the uncoated LPSCl surface. On the other hand, at a TMS‐SH concentration of 0.2 m, the coating layer of TMS‐SH was excessively thick, resulting in poor rate performance due to reduced ionic conductivity of TMS‐LPSCl.

### 2.3 Stable CEI Layer Derived from TMS‐SH

**Figure 7:**
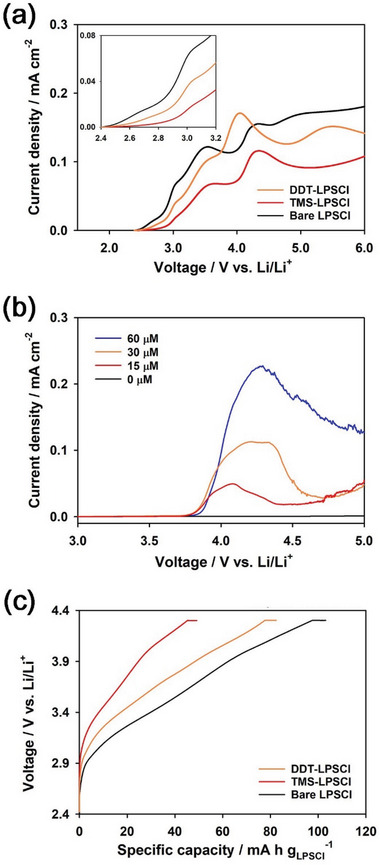
Formation of the CEI layer derived from TMS‐SH. a) LSV profiles of the LPSCl/C composite electrodes for bare LPSCl, TMS‐LPSCl, and DDT‐LPSCl at a scan rate of 0.2 mV s^−1^. The inset shows the LSV profiles expanded for clarity. b) LSV profiles of the electrolytes of 1.3 m LiPF_6_ in EC/EMC/DMC containing various concentrations of TMS‐SH at a scan rate of 0.2 mV s^−1^. c) Charge profiles of the LPSCl/C composite electrodes for bare LPSCl, TMS‐LPSCl, and DDT‐LPSCl at a current density of 0.022 mA cm^−2^.

To evidence the formation of the CEI layer derived from TMS‐SH, we compared the linear sweep voltammetry (LSV) profiles of the LPSCl/C composite electrodes consisting of only LPSCl and carbon additive at a scan rate of 0.2 mV s^−1^ for bare LPSCl, DDT‐LPSCl, and TMS‐LPSCl (**Figure** **7**a). For the preparation of the LPSCl/C electrodes, LPSCl powders and carbon additives (super P) were mixed in a weight ratio of LPSCl:super P = 7:3. Bare LPSCl and DDT‐LPSCl showed higher current density during oxidation than did TMS‐LPSCl. This implies that TMS‐LPSCl was oxidatively more stable than bare LPSCl and DDT‐LPSCl, revealing that the CEI layer derived from TMS‐SH suppressed the electrochemical oxidative decomposition of LPSCl. Note that TMS‐LPSCl and DDT‐LPSCl showed more intense current peaks at ca. 4.2 and 4.0 V (vs Li/Li^+^) in the LSV profiles, respectively, compared to bare LPSCl. This suggests that TMS‐SH and DDT‐SH were electrochemically decomposed at ca. 4.2 and 4.0 V (vs Li/Li^+^) during charge, respectively. This is supported by the LSV profiles of the electrolyte of 1.3 m LiPF_6_ in ethylene carbonate/ethyl‐methyl carbonate/dimethyl carbonate (EC/EMC/DMC, 3:4:3 as a volume ratio) containing various amounts of TMS‐SH, as shown in Figure 7b. Al and Li metal foils were used as the working and counter electrodes, respectively. As TMS‐SH was added to the electrolyte, a new oxidation peak appeared at ca. 4.2 V (vs Li/Li^+^) compared to bare electrolyte containing no TMS‐SH. The peak current at ca. 4.2 V (vs Li/Li^+^) increased with increasing the concentration of TMS‐SH. This suggests that TMS‐SH was electrochemically decomposed at ca. 4.2 V (vs Li/Li^+^), forming a passivation layer on the LPSCl surface. This is coincident with the LSV profile of the TMS‐LPSCl/C composite electrode in Figure 7a. We also compared the charge profiles of the LPSCl/C electrodes at a current density of 0.022 mA cm^−2^ for bare LPSCl, DDT‐LPSCl, and TMS‐LPSCl (Figure 7c). The charge capacity of the LPSCl/C electrodes corresponds to the amount of the irreversible electrochemical oxidative decomposition of LPSCl on the carbon surface.^[^ ^53^ ^]^ The charge capacity of TMS‐LPSCl was smaller than those of bare LPSCl and DDT‐LPSCl. This implies that TMS‐SH gave rise to the formation of the stable passivation layer on the LPSCl surface, thus suppressing the electrochemical degradation of LPSCl.

**Figure 8:**
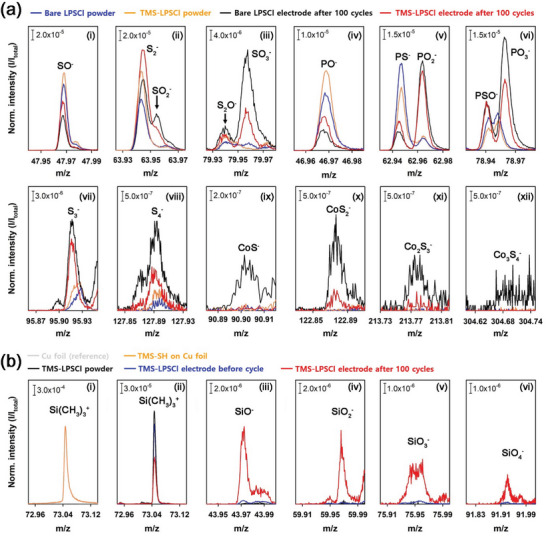
Improved interfacial stability of TMS‐LPSCl due to the CEI layer derived from TMS‐SH. a) Normalized TOF‐SIMS spectra of bare LPSCl and TMS‐LPSCl for negatively charged fragments. i–iii) Sulfur oxide (SO_x_^−^), iv–vi) phosphorous oxide (PO_x_^−^), vii,viii) polysulfide (S_x_^−^), and ix–xii) cobalt sulfide (Co_x_S_y_^−^) fragments. Blue, orange, black, and red spectra represent bare LPSCl powder, TMS‐LPSCl powder, bare LPSCl electrode after 100 cycles, and TMS‐LPSCl electrode after 100 cycles, respectively. b) Normalized TOF‐SIMS spectra of the composite cathode before and after cycling for TMS‐LPSCl, focusing on positively and negatively charged fragments, such as i,ii) trimethylsilyl group and iii–vi) silicon oxides. Grey, orange, black, blue, and red spectra represent i) bare Cu foil, i) TMS‐SH adsorbed on Cu foil, ii–vi) TMS‐LPSCl powder, ii–vi) the composite cathode containing TMS‐LPSCl before cycling, and ii–vi) the composite cathode containing TMS‐LPSCl after 100 cycles, respectively. The scale bars of normalized intensity are inserted in the figures.

We also investigated the CEI layers for i) bare LPSCl and ii) TMS‐LPSCl to clarify the formation of the stable CEI layer derived from TMS‐SH. The interface between LiCoO_2_ and LPSCl is known to be degraded during cycling because of the mutual diffusion of Co, O, P, and S between LiCoO_2_ and LPSCl, forming an unstable passivation layer containing cobalt sulfides, sulfates, and phosphates.^[^ ^23^, ^26^, ^31^ ^]^ Since cobalt sulfide is electrically conductive, the passivation layer containing cobalt sulfide is a mixed ion‐electron conductor.^[^ ^31^ ^]^ For this reason, the passivation layer grows gradually during cycling, leading to a gradual increase in the interfacial resistance between LiCoO_2_ and LPSCl. In this regard, we performed time‐of‐flight secondary‐ion mass spectrometry (TOF‐SIMS) and XPS analyses to compare the stability of interfacial passivation layers for bare LPSCl and TMS‐LPSCl. First of all, using TOF‐SIMS, we compared changes in the relative amounts of passivation layers at the interface during cycling for bare LPSCl and TMS‐LPSCl (**Figure** **8**; Figure S19, Supporting Information). The relative amounts of the constitutive components in the passivation layer were estimated from the normalized peak intensities of each species, such as the intensity of each fragment divided by the total intensity of all fragments including non‐oxidized species of LPSCl.^[^ ^57^, ^58^ ^]^ Figure 8a shows the ex situ TOF‐SIMS spectra of bare LPSCl and TMS‐LPSCl before and after cycling. The oxygen‐deficient SO_x_^−^ and PO_x_^−^ fragments, such as SO^−^ and PO^−^, were predominantly observed for pristine bare LPSCl and TMS‐LPSCl powders. However, the signal intensities of SO^−^ and PO^−^ for TMS‐LPSCl were slightly more intense than those for bare LPSCl because polar THF solvent slightly oxidized the LPSCl surface during synthesis, which is consistent with their XPS spectra (Figure 3; Figure S13, Supporting Information). After 100 cycles, however, the amounts of more oxidized SO_x_^−^ and PO_x_^−^ fragments (2 ≤ x ≤ 3) increased, whereas the amounts of the oxygen‐deficient SO^−^ and PO^−^ fragments decreased. This implies that the LPSCl surface was more oxidized during cycling due to its chemical and electrochemical reactions with LiCoO_2_, thus leading to severe interfacial degradation between LPSCl and LiCoO_2_.^[^ ^57^ ^]^ In addition, bare LPSCl showed higher increases in the signal intensities of more oxidized SO_x_^−^ and PO_x_^−^ fragments (2 ≤ x ≤ 3) after cycling compared to TMS‐LPSCl. The similar behavior was also observed for S_x_^−^ fragments (3 ≤ x ≤ 4). Moreover, in contrast to bare LPSCl, TMS‐LPSCl significantly suppressed the formation of cobalt sulfide compounds during cycling. This implies that the stable CEI layer derived from TMS‐SH mitigated the mutual atomic diffusion between LiCoO_2_ and LPSCl, eventually suppressing the gradual decomposition of LPSCl during cycling. Figure 8b compares the normalized TOF‐SIMS spectra of the composite cathode before and after cycling for TMS‐LPSCl, focusing on trimethylsilyl (Si(CH_3_)_3_^+^) and silicate (SiO_x_^−^ with 1 ≤ x ≤ 4) fragments. We observed only a trimethylsilyl (Si(CH_3_)_3_^+^) fragment for the composite cathode of TMS‐LPSCl and LiCoO_2_ before cycling, without any Si‐based chemical constituents, whereas the Si(CH_3_)_3_^+^ fragment was not observed on the bare LPSCl surface (Figure S19, Supporting Information). This reveals that TMS‐SH was not chemically decomposed on the LPSCl surface before cycling. Remarkably, however, various silicate (SiO_x_^−^ with 1 ≤ x ≤ 4) fragments were observed for TMS‐LPSCl after 100 cycles. This implies that TMS‐SH was electrochemically decomposed during cycling, forming a silicate‐based CEI layer. Moreover, the formation of the silicate‐based CEI layer reveals that the electrochemical decomposition of TMS‐SH occurred on the LiCoO_2_ surface because oxygen can only be obtained from LiCoO_2_. The formation of the stable CEI layer on the LiCoO_2_ surface due to TMS‐SH was also supported by ex situ XPS analysis. The S 2p, P 2p, and Si 2p XPS spectra of LPSCl powders and the composite cathodes at various cycle numbers for bare LPSCl and TMS‐LPSCl are presented in Figure S20 (Supporting Information). The XPS peaks (yellow) at 161.4 and 131.9 eV correspond to the S 2p and P 2p signals of the PS_4_^3−^ tetrahedra (P−S−Li bonds) in the argyrodite LPSCl structure, respectively. In addition, the red, brown, and blue peaks represent the decomposed species of LPSCl at the interface, such as polysulfide (‐S‐S‐), sulfate (SO_4_^2−^), and sulfite (SO_3_^2−^), respectively.^[^ ^59^ ^]^ Pristine TMS‐LPSCl powders were slightly more oxidized than bare LPSCl surface, which is coincident with the TOF‐SIMS results shown in Figure 8a. However, after cycling, bare LPSCl showed more severe interfacial degradation than did TMS‐LPSCl. The oxidized species of bare LPSCl significantly increased with increasing cycle numbers (Figure S20a,c), whereas the oxidation of TMS‐LPSCl was insignificant during cycling (Figure S20b,d,e). This implies that TMS‐SH gave rise to the formation of the stable CEI layer, thus suppressing the degradation of LPSCl at the interface during cycling.

### 2.4 Chemical Stability of LPSCl in a Dry Oxygen Atmosphere

**Figure 9:**
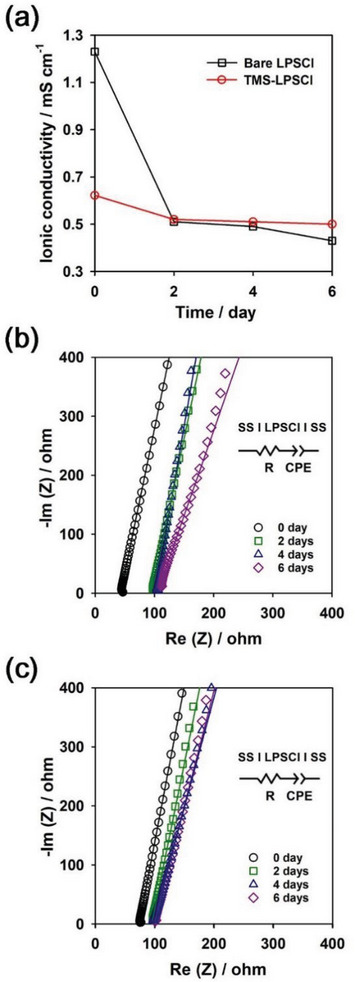
Chemical stability of LPSCl in a dry oxygen atmosphere. a) Changes in the ionic conductivity. b,c) The corresponding Nyquist plots of b) bare LPSCl and c) TMS‐LPSCl during storage in a dry oxygen atmosphere for six days. The symbols and linear lines in the Nyquist plots represent the EIS raw data and the fitted linear regression lines, respectively. The insets show the equivalent circuit model for fitting the Nyquist plots (SS: stainless steel, R: resistance, CPE: constant phase element).

Considering a cell assembly process for manufacturing ASSBs in industry, the chemical stability of LPSCl against oxygen gas in dry air is one of the important factors determining the electrochemical performance of ASSBs.^[^ ^6^, ^17^ ^]^ In this regard, we examined the chemical stability of LPSCl in a dry oxygen atmosphere. Both bare LPSCl and TMS‐LPSCl powders were simultaneously stored in a single container connected with inlet and outlet tubes at room temperature, as shown in Figure S21 (Supporting Information). Bare LPSCl and TMS‐LPSCl powders were separated using two vials in the container. Dry oxygen gas was flowed through the container at a flow rate of 100 ml min^−1^. LPSCl powders were stored in a container for various periods, such as 2, 4, and 6 days. Each sample set was stored in each batch container. After storage for various periods, samples were taken out from each container, followed by pelletizing the powders. The ionic conductivity of samples was then measured using EIS at room temperature. **Figure** **9**a shows changes in the ionic conductivity of bare LPSCl and TMS‐LPSCl as a function of time during storage in a dry oxygen atmosphere. The corresponding Nyquist plots of LPSCl and the fitted parameters were presented in Figure 9b,c, and Table S8 (Supporting Information), respectively. The ionic conductivity of bare LPSCl decreased significantly from 1.23 to 0.40 mS cm^−1^ after exposure to dry oxygen gas for 6 days. However, remarkably, the ionic conductivity of TMS‐LPSCl remained almost unchanged during exposure to dry oxygen gas even for 6 days. This was attributed to the fact that the TMS‐SH layer adsorbed on the LPSCl surface mitigated the chemical oxidation of LPSCl in a dry oxygen atmosphere, thereby improving the chemical stability of LPSCl with oxygen gas. In addition, we compared the electrochemical performances of bare LPSCl and TMS‐LPSCl powders retrieved after exposure to dry oxygen (Figure S22, Supporting Information). TMS‐LPSCl delivered a higher discharge capacity than did bare LPSCl. TMS‐LPSCl showed stable capacity retention even after the storage in dry oxygen. This reveals that TMS‐SH effectively suppressed the surface oxidation of LPSCl during the storage. In this regard, the TMS‐SH additive is promising for thiophosphate‐based solid electrolytes in terms of not only electrochemical performance but also practicality in industry.

## 3 Conclusion

We introduced the trimethylsilyl functional group‐based solid electrolyte additives for ASSBs to improve the interfacial stability of solid electrolytes. Trimethylsilyl compounds were examined as solid electrolyte additives because liquid electrolyte additives containing a trimethylsilyl group were known to form stable CEI layers on the cathode surface for Li‐ion batteries with liquid electrolytes. In addition, thiol compounds, such as TMS‐SH, were selected to deposit solid electrolyte additives on the LPSCl surface uniformly and thinly. This was because the hydrogen bonding and chalcogen–chalcogen interaction between thiol and thiophosphate gave rise to the molecular surface adsorption of TMS‐SH on the LPSCl surface. Consequently, the thin TMS‐SH layer, which was approximately a few nm in thickness, was uniformly adsorbed on the LPSCl surface.

TMS‐SH was oxidatively decomposed at ca. 4.2 V (vs Li/Li^+^), forming a stable silicate‐based CEI layer. This stable CEI layer suppressed the chemical and electrochemical degradation of the interface between LPSCl and LiCoO_2_ during cycling, which was evidenced by various ex situ XPS, TOF‐SIMS, and electrochemical analyses. Eventually, TMS‐SH gave rise to the excellent electrochemical performance of Li | LPSCl | LiCoO_2_, such as superior cycle life over 2000 cycles (85.0% of capacity retention after 2000 cycles) and high Coulombic efficiency (>99.9% for 2000 cycles). In addition, TMS‐SH improved the chemical stability of LPSCl in a dry oxygen atmosphere compared to bare LPSCl. Since the cell assembly of ASSBs in industry is carried out in a dry air atmosphere, the TMS‐SH additive showed promise not only in their electrochemical performance but also in their practicality in industry. These findings provide insight for developing practical thiophosphate‐based solid electrolytes for ASSBs.

## 4 Experimental Section

### Materials

For the preparation of LPSCl powders adsorbed with trimethylsilyl compounds, such as 2‐(trimethylsilyl)ethanethiol (Aldrich, 95%), 2‐(trimethylsilyl)ethanol (Aldrich, 96%), and bis(trimethylsilyl)methane (Aldrich, 97%), trimethylsilyl compounds were dissolved in THF (Alfa, 99.8%) solvent to obtain the solutions of 0.1 m trimethylsilyl compounds in THF. Commercial LPSCl powders were then added into the solutions at a weight ratio of LPSCl:trimethylsilyl compounds = 10:1, followed by stirring at 600 rpm for three hours to adsorb trimethylsilyl compounds on the LPSCl surface. LPSCl powders adsorbed with trimethylsilyl compounds were centrifuged to remove the THF solvent containing free trimethylsilyl compounds, followed by drying in a vacuum at 40 °C for 12 h. LPSCl powders adsorbed with 1‐dodecanethiol (Aldrich, 98%) were also prepared using the same procedure as LPSCl powders adsorbed with trimethylsilyl compounds. LPSCl powders exposed to THF solvent were obtained using the same procedure as for the synthesis of TMS‐LPSCl except for the addition of additives. LiNbO_3_‐coated LiCoO_2_ particles were synthesized using a conventional sol–gel method. Lithium ethoxide (Aldrich, 99.8%) and niobium ethoxide (Aldrich, 99%) were dissolved in anhydrous ethanol (Aldrich, 200 proof). LiCoO_2_ powders were stirred in the solution for 2 h, followed by evaporating ethanol using a rotary evaporator. The resulting powders were heated in an oxygen atmosphere at 450 °C for 3 h. Bare LiCoO_2_, LiNbO_3_‐coated LiCoO_2_, and carbon additives (super P) were dried in a vacuum oven at 120 °C overnight prior to cell assembly. A lithium metal foil (100 µm in thickness, Honzo, Japan) was used as an anode electrode. All chemicals were stored in an argon‐filled glovebox (<0.5 ppm O_2_, <0.1 ppm H_2_O)

### Material Characterizations

XRD patterns were acquired using a Bruker D2 PHASER with Cu Kα radiation (*λ* = 1.5418 Å) operated in the 2θ range of 10−60° for LPSCl and 10−80° for LiCoO_2_. An air‐tight cell with a beryllium window was used to avoid air exposure of LPSCl during XRD analysis. SEM and EDS mapping images were obtained using a field emission scanning electron microscope (Carl Zeiss, AURIGA, Germany). TEM and EDS mapping images were acquired using a field emission transmission electron microscope (JEOL, JEM‐F200 (TFEG), Japan). Cross‐sectional specimens were prepared with a focused ion beam (FIB) milling (JEOL Ltd., JEM‐F200). FT‐IR spectrometer (Bruker, TENSOR27) and RAMAN spectrometer II (DXR2xi) were used to collect IR and Raman spectra, respectively. An air‐tight cell was used to examine air‐sensitive LPSCl powders for Raman analysis. ICP‐AES analysis (OPTIMA 8300, Perkin‐Elmer, USA) was performed to measure the amount of TMS‐SH adsorbed on the LPSCl surface. LPSCl powders (0.03 g) were completely dissolved in 10 mL of deionized water using a vortex mixer with a polyethylene vial, because LPSCl powders are highly soluble in water. The resulting aqueous solution of LPSCl was then diluted with a 1% v/v HNO_3_ solution. Finally, the diluted solutions were analyzed for ICP‐AES. XPS analysis (SIGMA PROBE, Thermo Fisher Scientific, UK) was performed with monochromatic Al Kα radiation (1486.6 eV). The beam voltage and current were set to 15 kV and 7 mA, respectively. For ex situ XPS analysis, an XPS instrument was used equipped with a glovebox to avoid air exposure. All XPS spectra were calibrated using a signal of carbon at 284.8 eV. TOF‐SIMS analysis was performed in a negative and positive ion mode using a TOF.SIMS 5 instrument (ION‐TOF, Germany) with a 30 keV Bi^+^ primary ion source. All samples were transferred from a glovebox to the analysis chamber using a transfer vessel (ION‐TOF, Germany) to avoid air exposure. The transfer vessel was double‐sealed using a pouch cell to perform TOF‐SIMS analysis under more stringent air leakage‐free conditions. The analyses were run until a dose density limit of 1.0 × 10^13^ ions cm^−2^ was reached for the analysis area of 100 × 100 µm^2^.

### Fabrication of Bulk‐Type Cells

The electrochemical performance of Li | LPSCl | LiCoO_2_ was evaluated using a bulk‐type cell fabricated with the tri‐layers of a composite cathode pellet, a solid electrolyte pellet, and a Li metal foil. The schematic image of the bulk‐type cell is presented in Figure S6 (Supporting Information). The solid electrolyte layer pellet was prepared using bare LPSCl powders by pressing at 145 MPa. The LPSCl pellets were ca. 720 µm in thickness and 1.3 cm in diameter, as shown in Figure S23 (Supporting Information). For the preparation of the composite cathode pellets, LPSCl powders were mixed with LiCoO_2_ and carbon additives (super P) powders with a weight ratio of LiCoO_2_:LPSCl:super P = 12:7:1. The mixed powders were spread on the solid electrolyte layer pellet, followed by pressing at 360 MPa. The composite cathode pellets were ca. 50 µm in thickness and 1.3 cm in diameter. The mass loading of LiCoO_2_ was ≈6.8 mg cm^−2^. A Li metal foil (100 µm in thickness) was then attached to the other side of the solid electrolyte layer pellet. Then, the bulk‐type cells were assembled with a torque of 5 N m, which is equivalent to a stacking pressure of ≈41.4 MPa. The stacking pressure of the full cells was measured using a pressure sensor with a resolution of 0.1 kg (load cell, BONGSHIN). The cells remained without applying additional pressure during cycling. LiNbO_3_‐coated LiCoO_2_ was also assembled using the same procedure as bare LiCoO_2_.

### Electrochemical Cell Performance

Cycle performance was evaluated using TOSCAT‐3100 (TOYO, Japan). i) For the evaluation of long‐term cycle performance in Figure 5a, the bulk‐type cells were charged and discharged at current densities of 0.11 and 1.1 mA cm^−2^, respectively, after precycling at 0.11 mA cm^−2^ for charge and discharge during 3 cycles in the voltage range of 2.5–4.3 V (vs Li/Li^+^) at 30 °C. Charge was carried out using a constant current/constant voltage (CC/CV) mode in which a cell voltage was held at 4.3 V (vsLi/Li^+^) until current density was decayed to 0.05 mA cm^−2^. ii) For the evaluation of cycle performance in Figures 5c and 6a, the bulk‐type cells were charged and discharged at the same current density of 0.11 mA cm^−2^ using the CC/CV mode in the voltage ranges of 2.5–4.5 V and 2.5–4.3 V (vs Li/Li^+^), respectively, at 30 °C. iii) For the evaluation of cycle performance at a high charge current density in Figure 5d and Figure S10 (Supporting Information), the bulk‐type cells were charged at the current densities of 0.44 and 0.66 mA cm^−2^, respectively. The cells were discharged at the current density of 1.1 mA cm^−2^, using the CC mode in the voltage range of 2.5–4.3 V (vs Li/Li^+^) at 30 °C. iv) For the evaluation of discharge rate performance in Figure 6c, the cells were discharged at various current densities but charged at the same current density of 0.11 mA cm^−2^ using the CC/CV mode in the voltage range of 2.5–4.3 V (vs Li/Li^+^).

### Electrochemical Characterization

Li^+^ ion conductivity of LPSCl was measured using AC impedance with a Li^+^ ion blocking symmetric cell of stainless steel foil | LPSCl | stainless steel foil at various temperatures from −10 to 80 °C. LPSCl pellets were prepared by cold‐pressing at 360 MPa. AC impedance was carried out after the cells were held at each temperature for 2 h in a temperature‐controlled chamber. The activation energy of LPSCl was calculated using the equation below.

(1) $$\sigma \;=\;A\;\exp\left(\frac{-E_{a}}{k_{B}T}\right)$$

where σ,  A,  *k_B_*, *E_a_*, and *T* is ionic conductivity, pre‐exponential constant, Boltzmann constant, activation energy, and temperature in Kelvin, respectively. The logarithm of ionic conductivity (log σ) was linearly proportional to the reciprocal of temperature (1000/ *T*). The activation energy was calculated from a slope of the best‐fit linear regression line. The goodness‐of‐fit (R^2^) values of all samples were ≈0.99. Electronic conductivity of LPSCl was also measured by a DC impedance using a symmetric cell of In foil | LPSCl | In foil (50 um, MTI Korea Co.) under 0.25 V bias. The LPSCl pellet was prepared by applying a pressure of 360 MPa, and In foils were attached to both sides of the LPSCl pellet. Then the symmetric cell was assembled with a torque of 10 N m to enhance the contact between LPSCl pellet and In foil. Electrochemical impedance spectroscopy (EIS) was performed using an SP‐150 potentiostat (Biologic, France). EIS analysis was carried out by applying 10 mV amplitude at room temperature. The Nyquist plots were fitted using the EC‐Lab software V11.10 with a combination randomize + simplex mode. Fitting was stopped manually after the goodness‐of‐fit ($\frac{\chi^{2}}{\left|Z\right|}$) was in the order of 10^−2^. For LSV and galvanostatic analyses, the LPSCl/C composite electrode was prepared using LPSCl and super P powders with a weight ratio of 7:3 at 145 MPa. The bulk‐type cells for the LPSCl/C electrode were assembled using the same procedure as Li | LPSCl | LiCoO_2_. LSV was performed using an SP‐150 potentiostat (Biologic) at a scan rate of 0.2 mV s^−1^. The galvanostatic characterization of the LPSCl/C electrode was performed at a current density of 0.022 mA cm^−2^ using a CC/CV mode in which a constant voltage was held at 4.3 V (vs Li/Li^+^) until current density was decayed to 0.011 mA cm^−2^. The LSV profiles are obtained of TMS‐SH in liquid electrolytes for the various concentrations of TMS‐SH using a 2032 coin cell consisting of the working electrode of Al metal foil, the counter electrode of Li metal foil, and the electrolyte of 1.3 m LiPF_6_ in EC/EMC/DMC (3:4:3 as a volume ratio, Soulbrain Co. Ltd.).

### Evaluation of Chemical Stability in a Dry Oxygen Atmosphere

Both bare LPSCl and TMS‐LPSCl powders were simultaneously stored in a single container connected with inlet and outlet tubes at room temperature, as shown in Figure S21 (Supporting Information). A molecular sieve was also added to the container to remove a trace amount of moisture in oxygen gas. Bare LPSCl and TMS‐LPSCl powders were separated using two vials in the container. Dry oxygen gas was flowed through the container at a flow rate of 100 mL min^−1^. LPSCl powders were stored in a container for various periods, such as 2, 4, and 6 days. Each sample set was stored in each batch container. After storage for various periods, samples were taken out from each container, followed by pelletizing the powders. Then, the ionic conductivity of samples were measured using AC impedance. The electrochemical performance was evaluated in the voltage range of 2.5–4.3 V (vs Li/Li^+^) at a current density of 0.11 mA cm^−2^ and 30 °C.

## Conflict of Interest

The authors declare no conflict of interest.

## Supporting information

Supporting Information

## Data Availability

The data that support the findings of this study are available from the corresponding author upon reasonable request.

## References

[1] L. Zhou , T.‐T. Zuo , C. Y. Kwok , S. Y. Kim , A. Assoud , Q. Zhang , J. Janek , L. F. Nazar , Nat. Energy 2022, 7, 83.

[2] L. Duchêne , R.‐S. Kühnel , E. Stilp , E. C. Reyes , A. Remhof , H. Hagemann , C. Battaglia , Energy Environ. Sci. 2017, 10, 2609.

[3] H.‐D. Lim , J.‐H. Park , H.‐J. Shin , J. Jeong , J. T. Kim , K.‐W. Nam , H.‐G. Jung , K. Y. Chung , Energy Storage Mater. 2020, 25, 224.

[4] J. Janek , W. G. Zeier , Nat. Energy 2023, 8, 230.

[5] C. Park , J. Lee , S. Lee , Y. J. Han , J. Kim , S.‐K. Jung , Adv. Energy Mater. 2023, 13, 2203861.

[6] R. Chen , Q. Li , X. Yu , L. Chen , H. Li , Chem. Rev. 2020, 120, 6820. 31763824 10.1021/acs.chemrev.9b00268

[7] Y. Kato , S. Hori , T. Saito , K. Suzuki , M. Hirayama , A. Mitsui , M. Yonemura , H. Iba , R. Kanno , Nat. Energy 2016, 1, 16030.

[8] B. Helm , L. M. Gronych , A. Banik , M. A. Lange , C. Li , W. G. Zeier , Phys. Chem. Chem. Phys. 2023, 25, 1169. 36519415 10.1039/d2cp04710a

[9] S. Yu , K. Kim , B. C. Wood , H.‐G. Jung , K. Y. Chung , J. Mater. Chem. A 2022, 10, 24301.

[10] Y. Liu , S. Wang , A. M. Nolan , C. Ling , Y. Mo , Adv. Energy Mater. 2020, 10, 2002356.

[11] D. H. S. Tan , Y.‐T. Chen , H. Yang , W. Bao , B. Sreenarayanan , J.‐M. Doux , W. Li , B. Lu , S.‐Y. Ham , B. Sayahpour , J. Scharf , E. A. Wu , G. Deysher , H. E. Han , H. J. Hah , H. Jeong , J. B. Lee , Z. Chen , Y. S. Meng , Science 2021, 373, 1494. 34554780 10.1126/science.abg7217

[12] T. Asano , A. Sakai , S. Ouchi , M. Sakaida , A. Miyazaki , S. Hasegawa , Adv. Mater. 2018, 30, 1803075. 10.1002/adma.201803075 30216562

[13] L. Duchêne , A. Remhof , H. Hagemann , C. Battaglia , Energy Storage Mater. 2020, 25, 782.

[14] Y.‐G. Lee , S. Fujiki , C. Jung , N. Suzuki , N. Yashiro , R. Omoda , D.‐S. Ko , T. Shiratsuchi , T. Sugimoto , S. Ryu , J. H. Ku , T. Watanabe , Y. Park , Y. Aihara , D. Im , I. T. Han , Nat. Energy 2020, 5, 299.

[15] A. Sakuda , A. Hayashi , M. Tatsumisago , Sci. Rep. 2013, 3, 2261. 23877241 10.1038/srep02261 PMC3719077

[16] Y. J. Nam , S.‐J. Cho , D. Y. Oh , J.‐M. Lim , S. Y. Kim , J. H. Song , Y.‐G. Lee , S.‐Y. Lee , Y. S. Jung , Nano Lett. 2015, 15, 3317. 25919229 10.1021/acs.nanolett.5b00538

[17] L. Xu , Y. Lu , C.‐Z. Zhao , H. Yuan , G.‐L. Zhu , L.‐P. Hou , Q. Zhang , J.‐Q. Huang , Adv. Energy Mater. 2021, 11, 2002360.

[18] T. Kim , K. Kim , S. Lee , G. Song , M. S. Jung , K. T. Lee , Chem. Mater. 2022, 34, 9159.

[19] C. Wang , J. T. Kim , C. Wang , X. Sun , Adv. Mater. 2022, 35, 2209074.

[20] H. Muramatsu , A. Hayashi , T. Ohtomo , S. Hama , M. Tatsumisago , Solid State Ionics 2011, 182, 116.

[21] J. Xu , Y. Li , P. Lu , W. Yan , M. Yang , H. Li , L. Chen , F. Wu , Adv. Energy Mater. 2022, 12, 2102348.

[22] J. Auvergniot , A. Cassel , J.‐B. Ledeuil , V. Viallet , V. Seznec , R. Dedryvère , Chem. Mater. 2017, 29, 3883.

[23] R. Koerver , I. Aygün , T. Leichtweiß , C. Dietrich , W. Zhang , J. O. Binder , P. Hartmann , W. G. Zeier , J. Janek , Chem. Mater. 2017, 29, 5574.

[24] J. Haruyama , K. Sodeyama , Y. Tateyama , ACS Appl. Mater. Interfaces 2017, 9, 286. 27991765 10.1021/acsami.6b08435

[25] A. Sakuda , A. Hayashi , M. Tatsumisago , Chem. Mater. 2010, 22, 949.

[26] W. Zhang , F. H. Richter , S. P. Culver , T. Leichtweiss , J. G. Lozano , C. Dietrich , P. G. Bruce , W. G. Zeier , J. Janek , ACS Appl. Mater. Interfaces 2018, 10, 22226. 29877698 10.1021/acsami.8b05132

[27] Y. Zhu , X. He , Y. Mo , ACS Appl. Mater. Interfaces 2015, 7, 23685. 26440586 10.1021/acsami.5b07517

[28] W. D. Richards , L. J. Miara , Y. Wang , J. C. Kim , G. Ceder , Chem. Mater. 2016, 28, 266.

[29] N. Ohta , K. Takada , I. Sakaguchi , L. Zhang , R. Ma , K. Fukuda , M. Osada , T. Sasaki , Electrochem. Commun. 2007, 9, 1486.

[30] S. Ito , S. Fujiki , T. Yamada , Y. Aihara , Y. Park , T. Y. Kim , S.‐W. Baek , J.‐M. Lee , S. Doo , N. Machida , J. Power Sources 2014, 248, 943.

[31] S. H. Jung , K. Oh , Y. J. Nam , D. Y. Oh , P. Brüner , K. Kang , Y. S. Jung , Chem. Mater. 2018, 30, 8190.

[32] S. P. Culver , R. Koerver , W. G. Zeier , J. Janek , Adv. Energy Mater. 2019, 9, 1900626.

[33] W. Zhang , T. Leichtweiß , S. P. Culver , R. Koerver , D. Das , D. A. Weber , W. G. Zeier , J. Janek , ACS Appl. Mater. Interfaces 2017, 9, 35888. 28937736 10.1021/acsami.7b11530

[34] T. Hakari , M. Deguchi , K. Mitsuhara , T. Ohta , K. Saito , Y. Orikasa , Y. Uchimoto , Y. Kowada , A. Hayashi , M. Tatsumisago , Chem. Mater. 2017, 29, 4768.

[35] S. Deng , Y. Sun , X. Li , Z. Ren , J. Liang , K. Doyle‐Davis , J. Liang , W. Li , M. Norouzi Banis , Q. Sun , R. Li , Y. Hu , H. Huang , L. Zhang , S. Lu , J. Luo , X. Sun , ACS Energy Lett. 2020, 5, 1243.

[36] S. Randau , F. Walther , A. Neumann , Y. Schneider , R. S. Negi , B. Mogwitz , J. Sann , K. Becker‐Steinberger , T. Danner , S. Hein , A. Latz , F. H. Richter , J. Janek , Chem. Mater. 2021, 33, 1380.

[37] Z. Yu , S.‐L. Shang , K. Ahn , D. T. Marty , R. Feng , M. H. Engelhard , Z.‐K. Liu , D. Lu , ACS Appl. Mater. Interfaces 2022, 14, 32035. 35816730 10.1021/acsami.2c07388

[38] Z. D. Hood , A. U. Mane , A. Sundar , S. Tepavcevic , P. Zapol , U. D. Eze , S. P. Adhikari , E. Lee , G. E. Sterbinsky , J. W. Elam , J. G. Connell , Adv. Mater. 2023, 35, 2300673. 10.1002/adma.202300673 36929566

[39] W. D. Jung , M. Jeon , S. S. Shin , J.‐S. Kim , H.‐G. Jung , B.‐K. Kim , J.‐H. Lee , Y.‐C. Chung , H. Kim , ACS Omega 2020, 5, 26015. 33073128 10.1021/acsomega.0c03453 PMC7558032

[40] A. L. Davis , R. Garcia‐Mendez , K. N. Wood , E. Kazyak , K.‐H. Chen , G. Teeter , J. Sakamoto , N. P. Dasgupta , J. Mater. Chem. A 2020, 8, 6291.

[41] J. Li , Y. Li , J. Cheng , Q. Sun , L. Dai , N. Ci , D. Li , L. Ci , J. Power Sources 2022, 518, 230739.

[42] I. Sasaki , K. Honda , T. Asano , Y. Ito , T. Komori , J. Hibino , Solid State Ionics 2020, 347, 115249.

[43] F. Wu , W. Fitzhugh , L. Ye , J. Ning , X. Li , Nat. Commun. 2018, 9, 4037. 30279498 10.1038/s41467-018-06123-2 PMC6168527

[44] J.‐G. Han , M.‐Y. Jeong , K. Kim , C. Park , C. H. Sung , D. W. Bak , K. H. Kim , K.‐M. Jeong , N.‐S. Choi , J. Power Sources 2020, 446, 227366.

[45] R. Fang , B. Xu , N. S. Grundish , Y. Xia , Y. Li , C. Lu , Y. Liu , N. Wu , J. B. Goodenough , Angew. Chem. 2021, 133, 17842. 10.1002/anie.202106039 34192402

[46] H. Rong , M. Xu , B. Xie , W. Huang , X. Liao , L. Xing , W. Li , J. Power Sources 2015, 274, 1155.

[47] Y. Liu , L. Tan , L. Li , J. Power Sources 2013, 221, 90.

[48] I. S. Antonijević , G. V. Janjić , M. K. Milčić , S. D. Zarić , Cryst. Growth Des. 2016, 16, 632.

[49] Y. Wen , S. Zuzhen , J. Junru , Z. Hongyang , H. Xinmei , H. Zhijun , Z. Jian , C. Qiliang , J. Phys. Chem. B 2021, 125, 12042. 34704437 10.1021/acs.jpcb.1c08492

[50] B. Chiavarino , M. E. Crestoni , J. Lemaire , P. Maitre , S. Fornarini , J. Chem. Phys. 2013, 139, 071102. 23968063 10.1063/1.4818729

[51] G. Liu , J. Shi , M. Zhu , W. Weng , L. Shen , J. Yang , X. Yao , Energy Storage Mater. 2021, 38, 249.

[52] Y. Zhao , Y. Yan , Z. Wu , C. Li , R. Fan , L. Feng , W. Wang , Q. Chen , J. Mater. Sci. 2022, 57, 13425.

[53] D. H. S. Tan , E. A. Wu , H. Nguyen , Z. Chen , M. A. T. Marple , J.‐M. Doux , X. Wang , H. Yang , A. Banerjee , Y. S. Meng , ACS Energy Lett. 2019, 4, 2418.

[54] J. R. Reimers , M. J. Ford , A. Halder , J. Ulstrup , N. S. Hush , Proc. Natl Acad. Sci. U. S. A. 2016, 113, E1424. 26929334 10.1073/pnas.1600472113 PMC4801306

[55] L. Zhou , Y. Zhao , H. Ma , J. Nanopart. Res. 2020, 22, 199.

[56] J. Woo , Y. B. Song , H. Kwak , S. Jun , B. Y. Jang , J. Park , K. T. Kim , C. Park , C. Lee , K. H. Park , Adv. Energy Mater. 2022, 13, 2203292.

[57] F. Walther , R. Koerver , T. Fuchs , S. Ohno , J. Sann , M. Rohnke , W. G. Zeier , J. Janek , Chem. Mater. 2019, 31, 3745.

[58] F. Walther , F. Strauss , X. Wu , B. Mogwitz , J. Hertle , J. Sann , M. Rohnke , T. Brezesinski , J. Janek , Chem. Mater. 2021, 33, 2110.

[59] C. Wang , S. Hwang , M. Jiang , J. Liang , Y. Sun , K. Adair , M. Zheng , S. Mukherjee , X. Li , R. Li , H. Huang , S. Zhao , L. Zhang , S. Lu , J. Wang , C. V. Singh , D. Su , X. Sun , Adv. Energy Mater. 2021, 11, 2100210.

