# Supplementary material for: Trimethylsilyl Compounds for the Interfacial Stabilization of Thiophosphate‐Based Solid Electrolytes in All‐Solid‐State Batteries
Source: Adv Sci (Weinh). 2023 Oct 22;10(33):2303308. doi: 10.1002/advs.202303308 (PMC10667807; doi:10.1002/advs.202303308)
Supplement: Supplementary file 1 — Supporting Information [file ADVS-10-2303308-s001.pdf]

ADVANCED SCIENCE
Open Access

Supporting Information

for *Adv. Sci.*, DOI 10.1002/advs.202303308

Trimethylsilyl Compounds for the Interfacial Stabilization of Thiophosphate-Based Solid Electrolytes in All-Solid-State Batteries

*Kanghyeon Kim, Taehun Kim, Gawon Song, Seonghyun Lee, Min Soo Jung, Seongmin Ha, A. Reum Ha and Kyu Tae Lee**

Supporting Information

Trimethylsilyl Compounds for the Interfacial Stabilization of Thiophosphate-based Solid Electrolytes in All-Solid-State Batteries

Kanghyeon Kim, Taehun Kim, Gawon Song, Seonghyun Lee, Min Soo Jung, Seongmin Ha, A Reum Ha, Kyu Tae Lee*

K. Kim, T. Kim, G. Song, S. Lee, M. S. Jung, K. T. Lee
School of Chemical and Biological Engineering, Institute of Chemical Processes, Seoul National University, 1 Gwanak-ro, Gwanak-gu, Seoul 08826, Republic of Korea.
E-mail: ktlee@snu.ac.kr

S. Ha, A. R. Ha
Advanced Battery Development Team 1, Hyundai Motor Company, 37 Cheoldobangmulgwan-ro, Uiwang-Si, Gyeonggi-do, 16082, Republic of Korea.

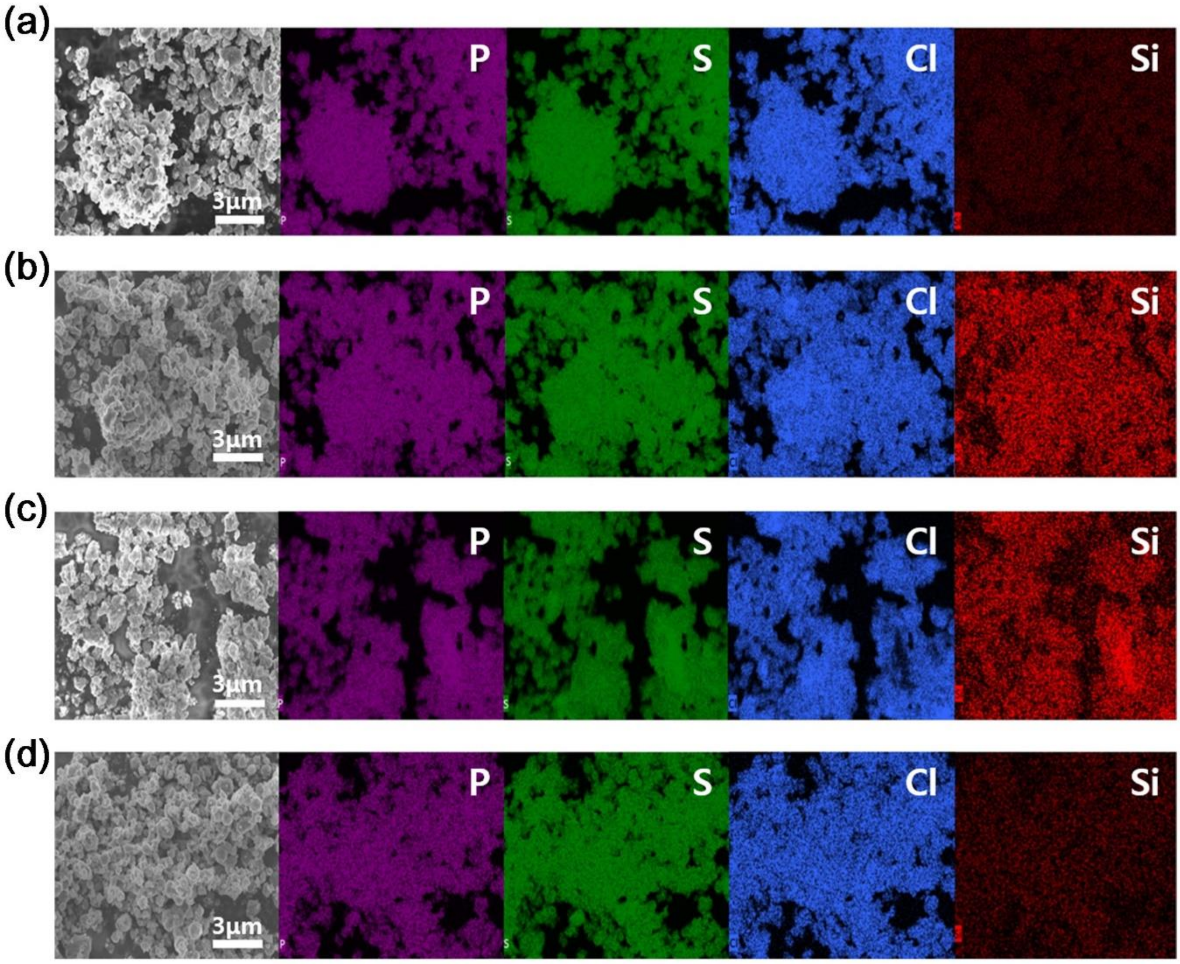

Figure S1. a-d) SEM and EDS mapping images of LPSCl powders adsorbed with solid electrolyte additives. Bare LPSCl powders (a) and LPSCl powders adsorbed with TMS-SH (b), TMS-OH (c), and Bis-TMS (d). Violet, green, blue, and red colors represent phosphorus, sulfur, chlorine, and silicon elements, respectively.

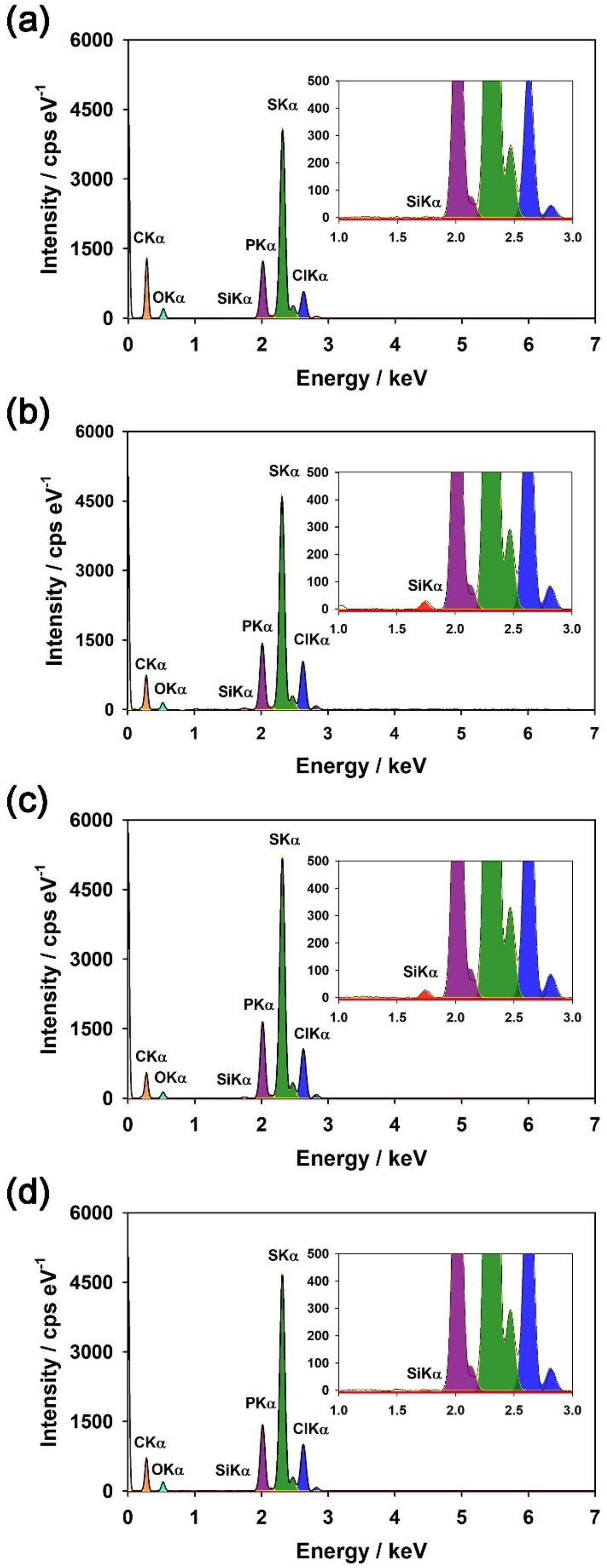

Figure S2. a-d) EDS spectra for bare LPSCl powders (a) and LPSCl powders adsorbed with solid electrolyte additives, such as TMS-SH (b), TMS-OH (c), and Bis-TMS (d). Horizontal and vertical axes in the EDS spectra represent energy (keV) and counts per seconds per electron-volt (cps eV^{-1}), respectively. The insets show the EDS spectra expanded for clarity.

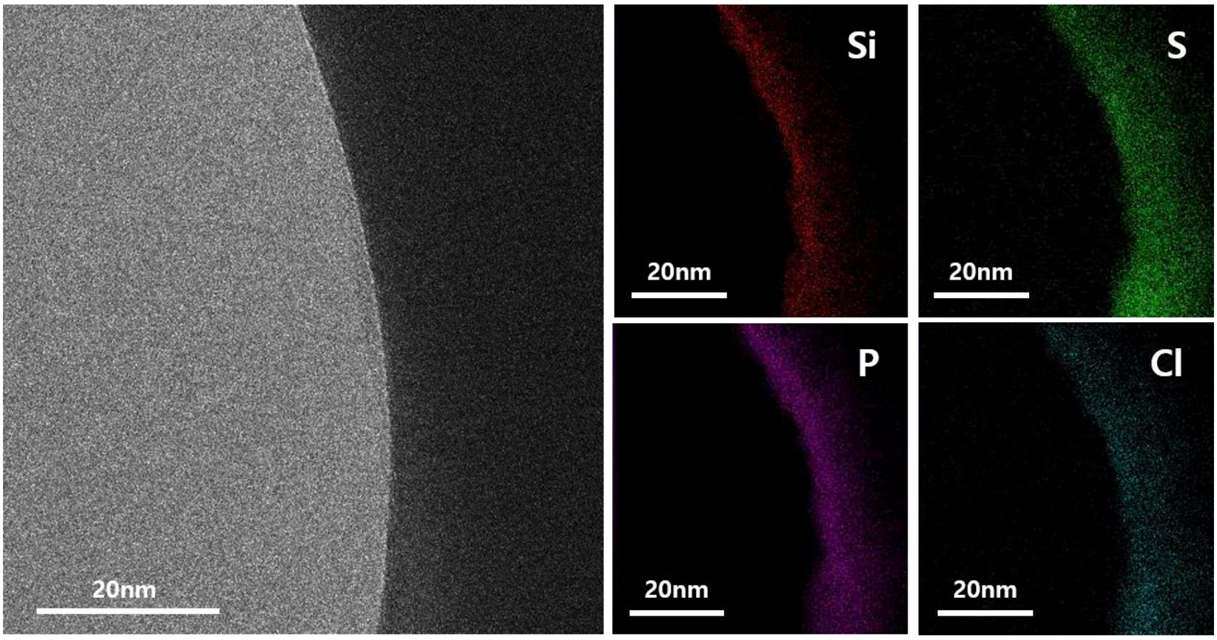

Figure S3. TEM and EDS mapping images of LPSCl powders adsorbed with TMS-OH. Red, green, violet, and blue colors represent silicon, sulfur, phosphorus, and chlorine elements, respectively.

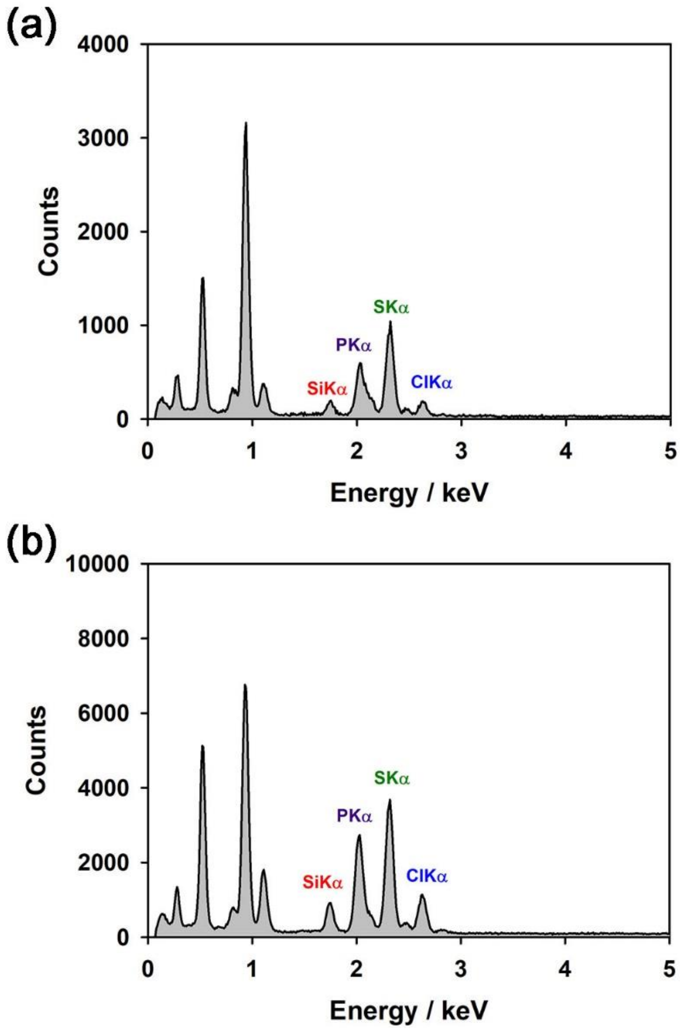

Figure S4. a, b) TEM-EDS spectra of LPSCl adsorbed with TMS-SH (a) and TMS-OH (b). Horizontal and vertical axes in the spectra represent energy (keV) and counts, respectively.

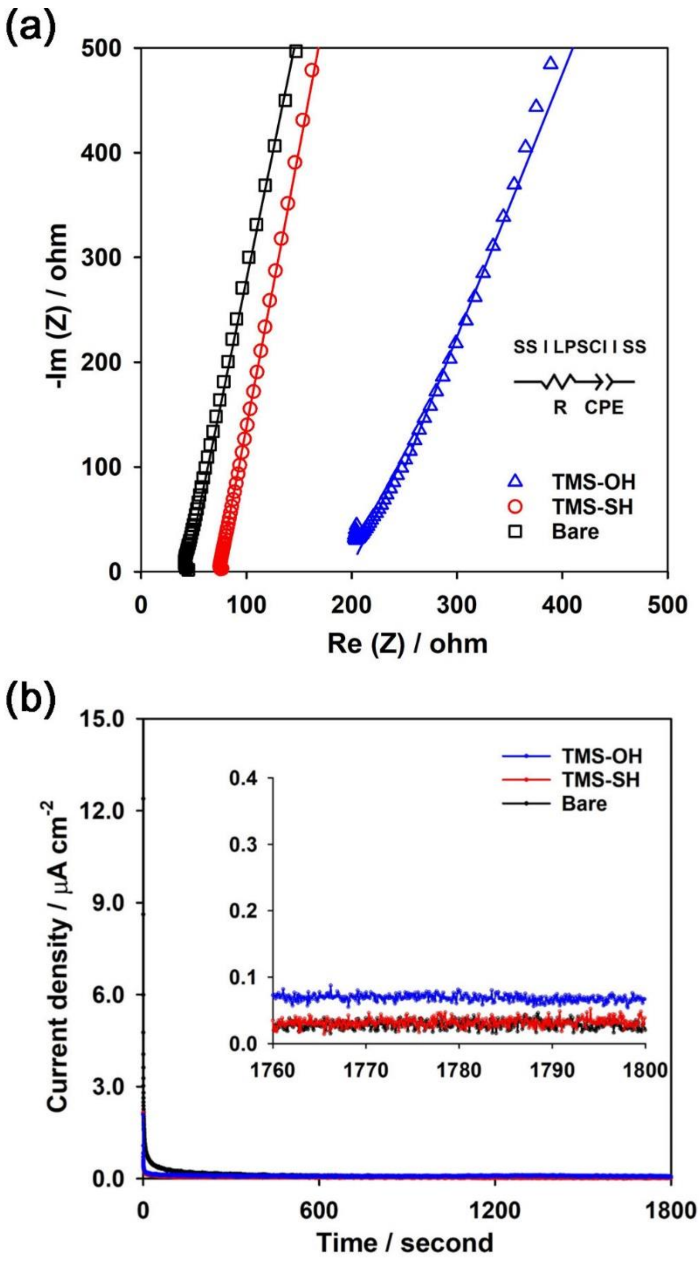

Figure S5. a) Nyquist plots and b) chronoamperometry profiles of bare LPSCl and LPSCl adsorbed with TMS-OH and TMS-SH under 0.25 V bias. Symbols and solid lines in (a) represent raw data and linear fit lines, respectively. The inset in (a) shows the equivalent circuit model for fitting the Nyquist plots (SS: stainless steel, R: resistance, and CPE: constant phase element). The inset in (b) shows the chronoamperometry profiles on an expanded y-axis scale in the selected time period for clarity. Bare, TMS-OH, and TMS-SH in the figure legends represent bare LPSCl, LPSCl adsorbed with TMS-OH, and LPSCl adsorbed with TMS-SH, respectively.

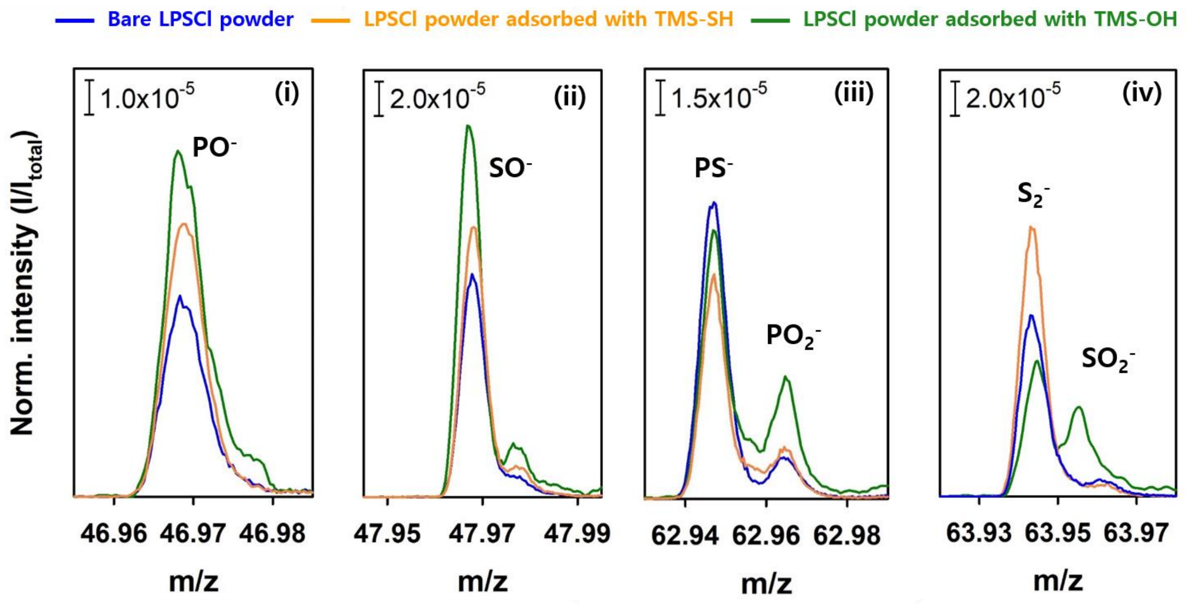

Figure S6. Normalized TOF-SIMS spectra of bare LPSCl power, LPSCl powder adsorbed with TMS-SH, and LPSCl powder adsorbed with TMS-OH for negatively charged fragments. Phosphorous oxide (PO_x^-) (i, iii), sulfur oxide (SO_x^-) (ii, iv), and polysulfide (S_2^-) (iv). Blue, orange, and green spectra represent bare LPSCl powder, LPSCl powder adsorbed with TMS-SH, and LPSCl powder adsorbed with TMS-OH, respectively. The scale bars of normalized intensity are inserted in the figures.

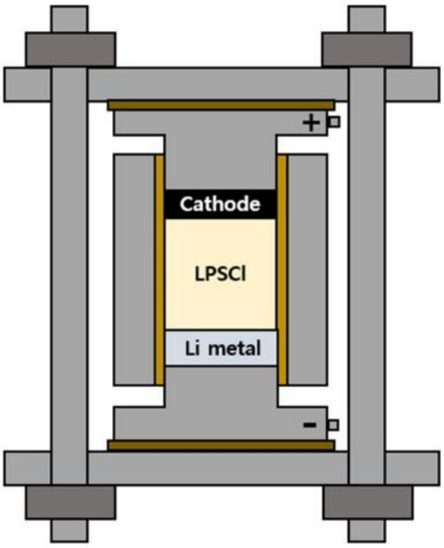

Figure S7. Schematic image of the home-made bulk-type cell.

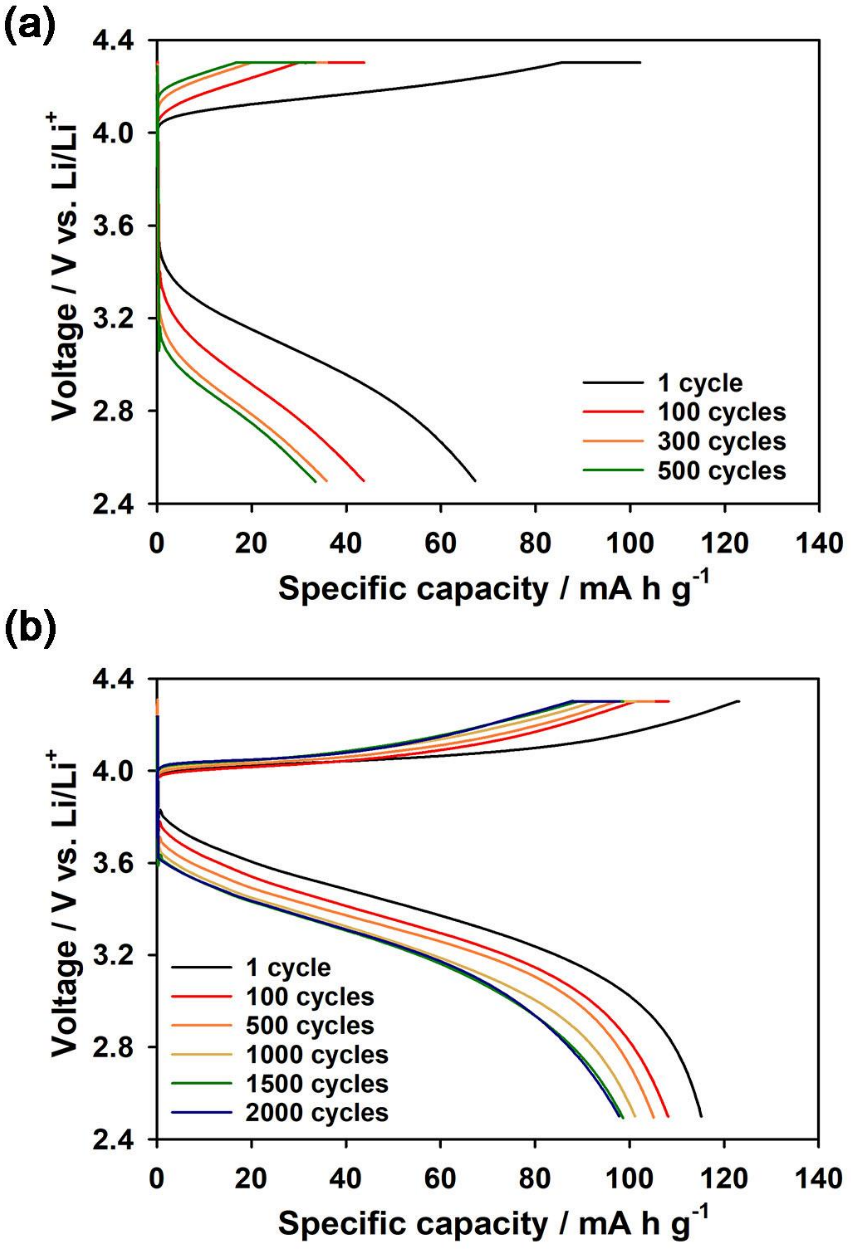

Figure S8. a, b) Voltage profiles of bare $LiCoO_2$ with bare LPSCl (Bare LCO / Bare LPSCl) (a) and bare $LiCoO_2$ with TMS-LPSCl (Bare LCO / TMS-LPSCl) (b) for various cycle numbers in the voltage range of 2.5 – 4.3 V (vs. Li/Li^+) at charge and discharge current densities of 0.11 and 1.1 mA cm^{-2}, respectively, after precycling at 0.11 mA cm^{-2}.

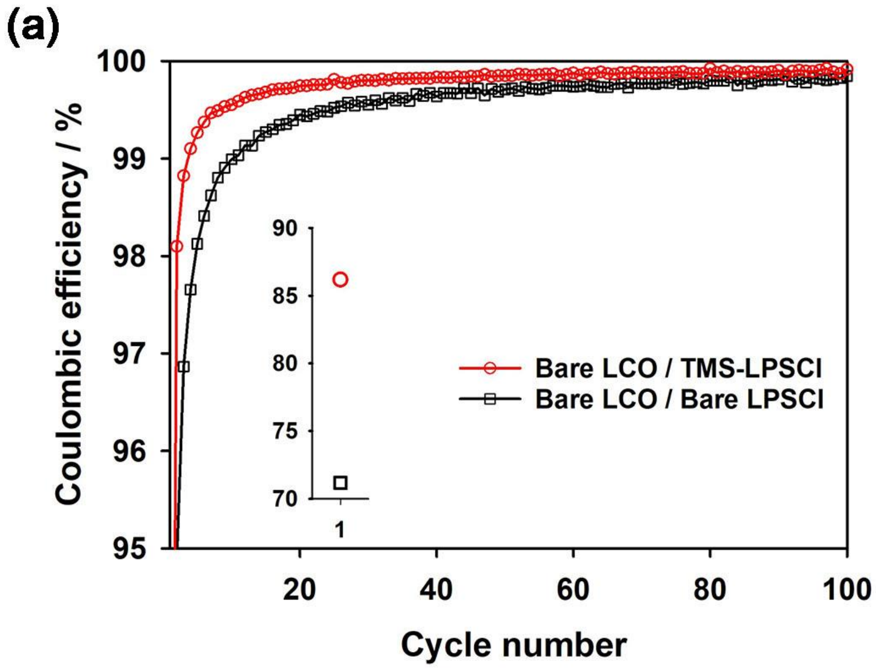

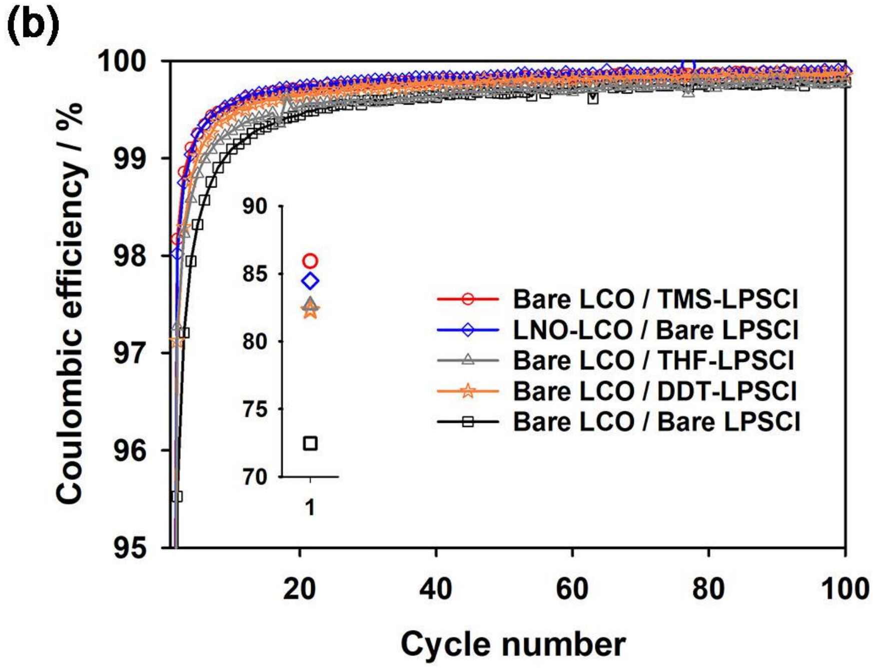

Figure S9. a, b) Coulombic efficiency of various electrochemical cells shown in Figure 4c (a) and Figure 5a (b). The inset shows their Coulombic efficiencies at the first cycle. $LiCoO_2$ and $LiNbO_3$ were denoted as LCO and LNO, respectively.

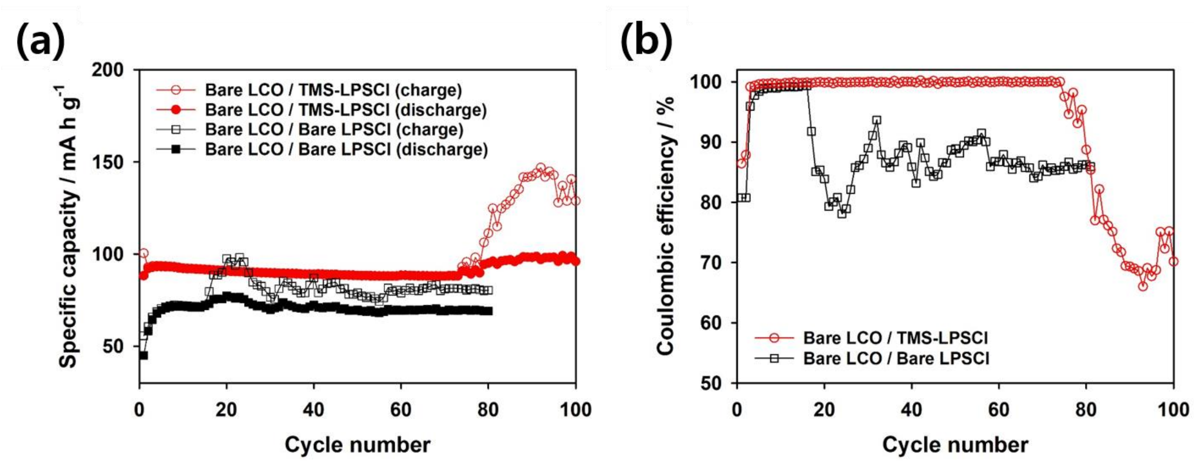

Figure S10. a) Cycle performances and b) Coulombic efficiencies of Li | LPSCl | $LiCoO_2$ cells at a charge current density of 0.66 mA cm^{-2} of bare LPSCl and TMS-LPSCl for the composite cathode pellets. The cells were discharged at the same current density of 1.1 mA cm^{-2} using a CC mode in the voltage range of 2.5 – 4.3 V (vs. Li/Li^+) at 30 °C.

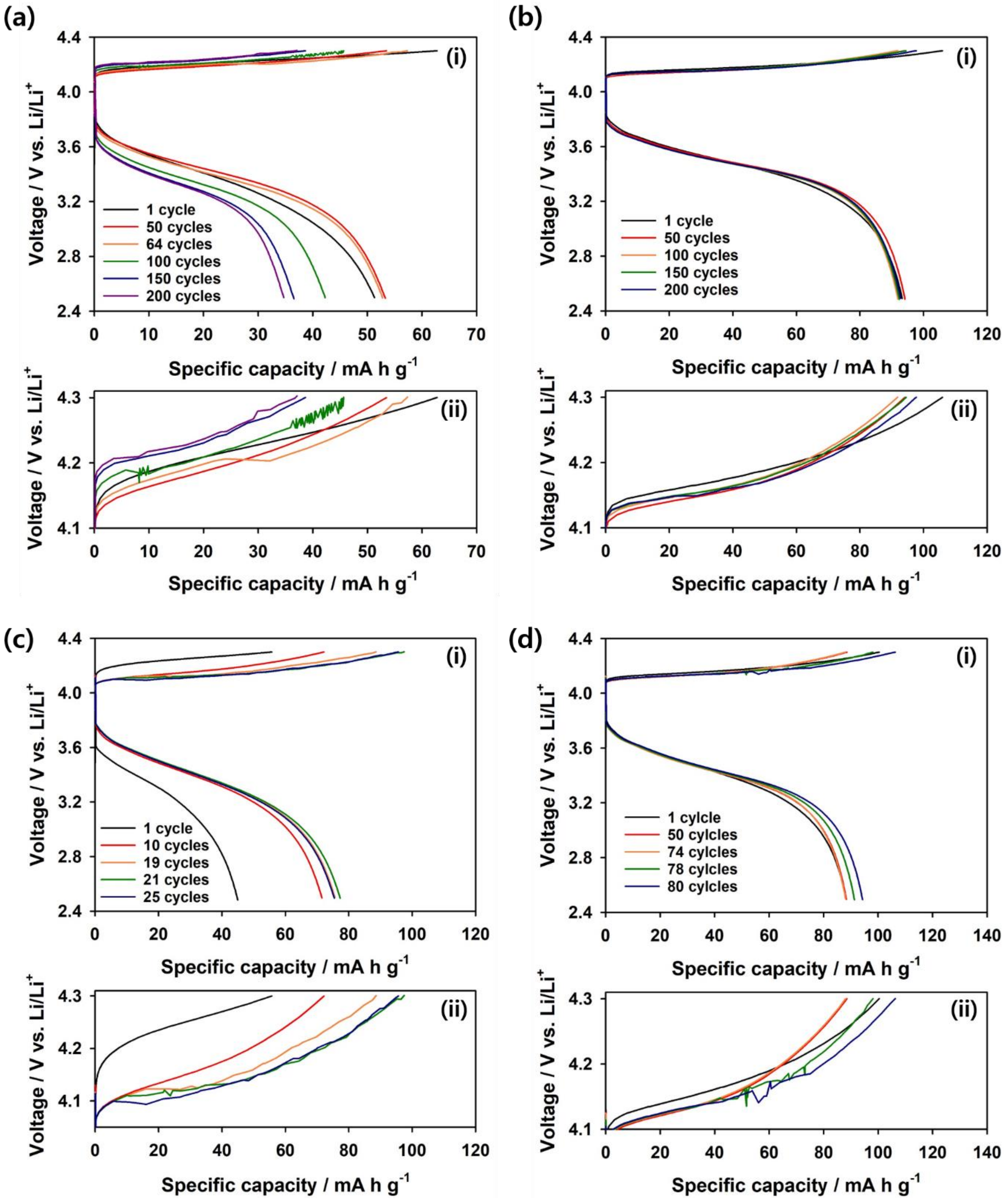

Figure S11. a-d) Voltage profiles of Li | LPSCl | $LiCoO_2$ cells at a various charge current densities, such as 0.44 mA cm^{-2} (a, b) and 0.66 mA cm^{-2} (c, d), for bare LPSCl (a, c) and TMS-LPSCl (b, d) in the composite cathode pellets. The cells were discharged at the same current density of 1.1 mA cm^{-2} using a CC mode in the voltage range of 2.5 – 4.3 V (vs. Li/Li^+) at 30 °C. To clearly display the voltage fluctuation in the end of charge due to micro-short circuit, the voltage profiles of the selected voltage range of 4.1 − 4.3 V in (i) were displayed in (ii).

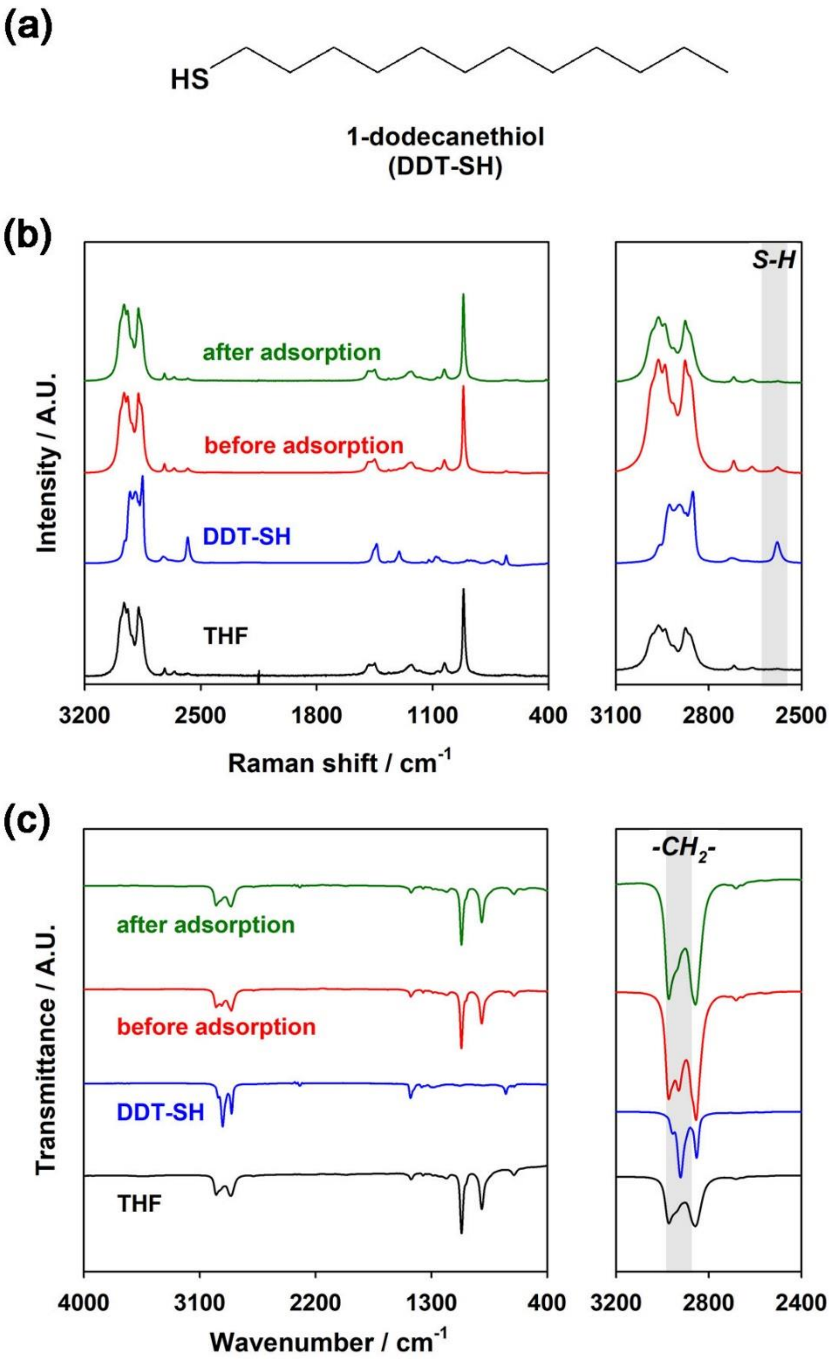

Figure S12. a) 2D molecular structure of DDT-SH. b) Raman and c) IR spectra of THF solutions containing DDT-SH before and after adsorption of DDT-SH on the LPSCl surface. The regions of the Raman (2500 – 3100 cm^{-1}) and IR (3200 − 2400 cm^{-1}) spectra are expanded for clarity.

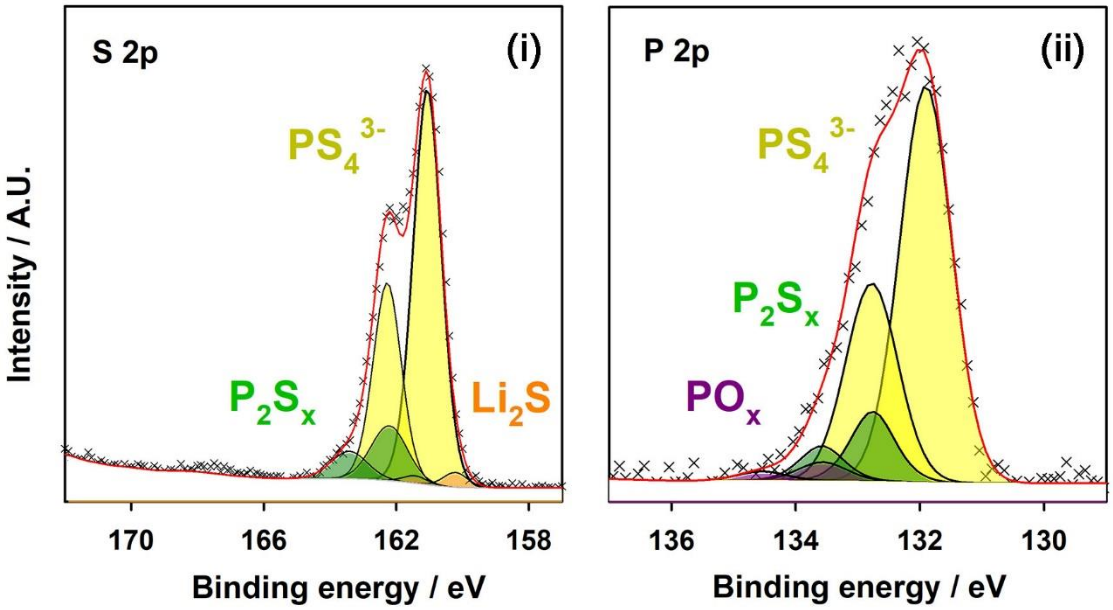

Figure S13. S 2p and P 2p XPS spectra of THF-LPSCl powders.

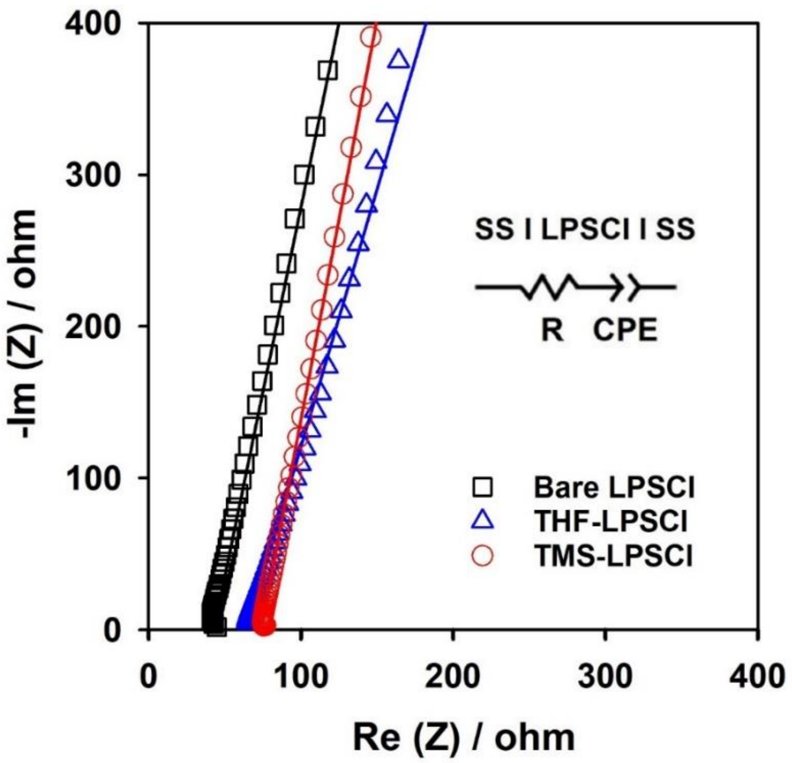

Figure S14. Nyquist plots of bare LPSCl (black square), THF-LPSCl (blue triangle), and TMS-LPSCl (red circle). Symbols and solid lines represent raw data and linear fit lines, respectively. The inset shows the equivalent circuit model for the Nyquist plots (SS: stainless steel, R: resistance, and CPE: constant phase element).

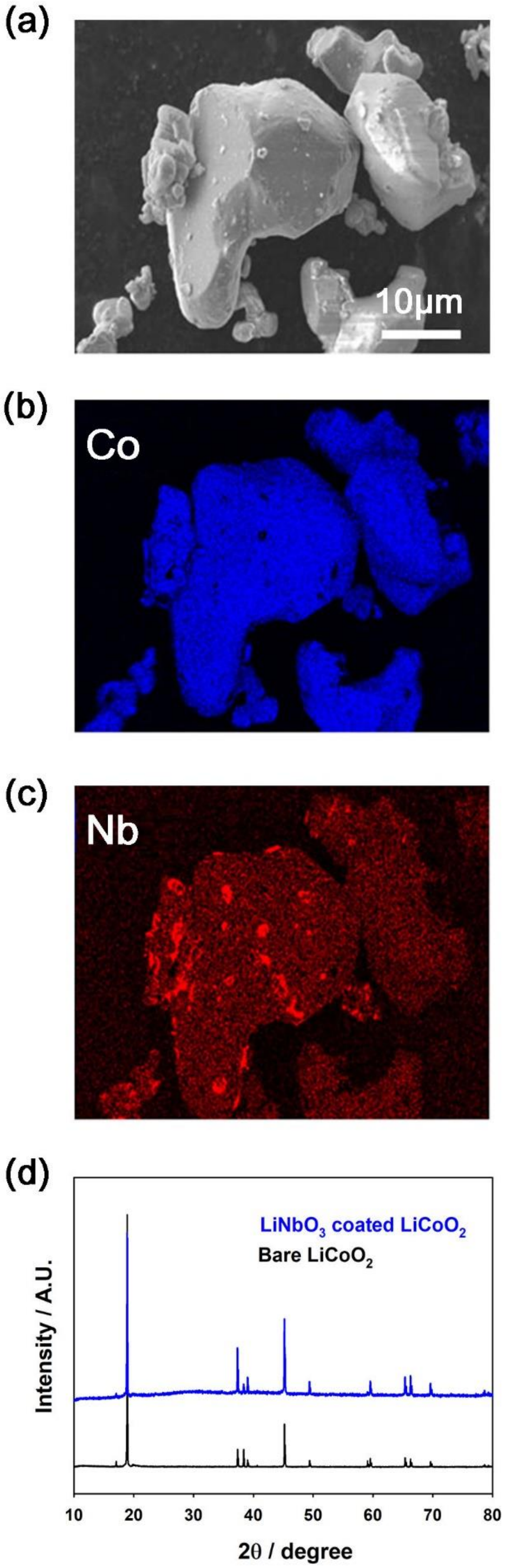

Figure S15. a-c) SEM and EDS mapping images of $LiNbO_3$-coated $LiCoO_2$. d) XRD patterns of bare $LiCoO_2$ and $LiNbO_3$-coated $LiCoO_2$.

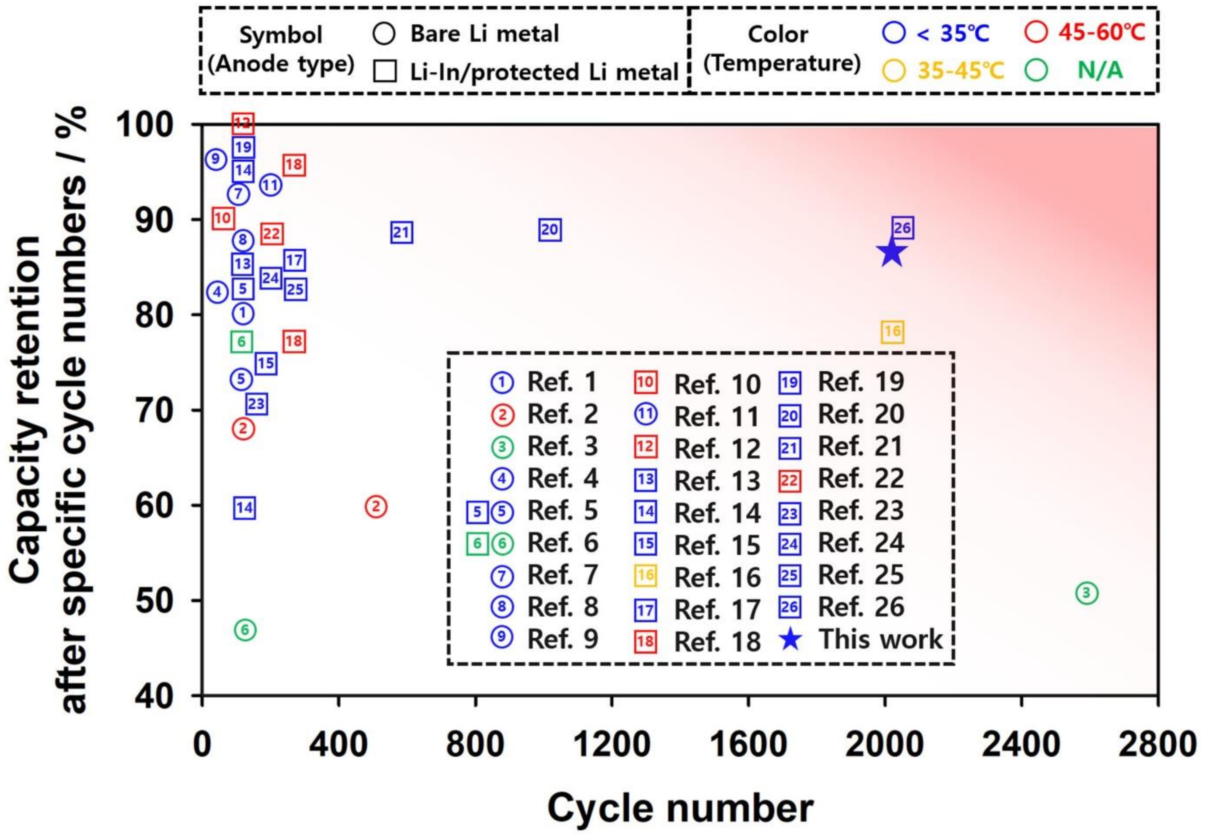

Figure S16. Comparison of the cycle performances of $LiCoO_2$ cathode materials with sulfide-based solid electrolytes reported in recent literatures. Symbols and colors represent anode type and operating temperature, respectively: circle (bare Li metal), square (protected Li metal such as Li-In and coated Li), blue (25 ~ 35 ℃ including room temperature), orange (35 ~ 45 ℃), red (45 ~ 60 ℃), and green (N/A). The detailed cell conditions are listed in Table S5.

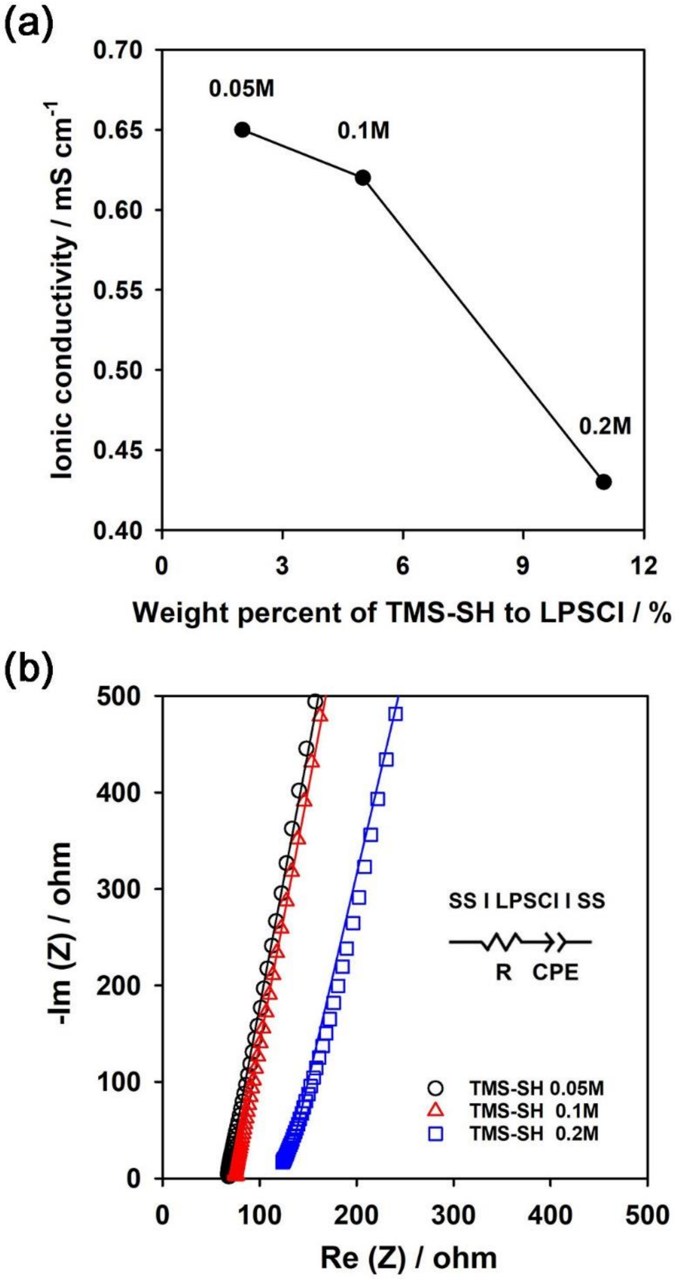

Figure S17. a, b) Ionic conductivities (a) and the corresponding Nyquist plots (b) of LPSCl adsorbed with TMS-SH for various concentrations of TMS-SH in THF. The weight percent of TMS-SH to LPSCl was measured using ICP-AES analysis. Symbols and solid lines represent raw data and linear fit lines, respectively. The inset shows the equivalent circuit model for the Nyquist plots (SS: stainless steel, R: resistance, and CPE: constant phase element).

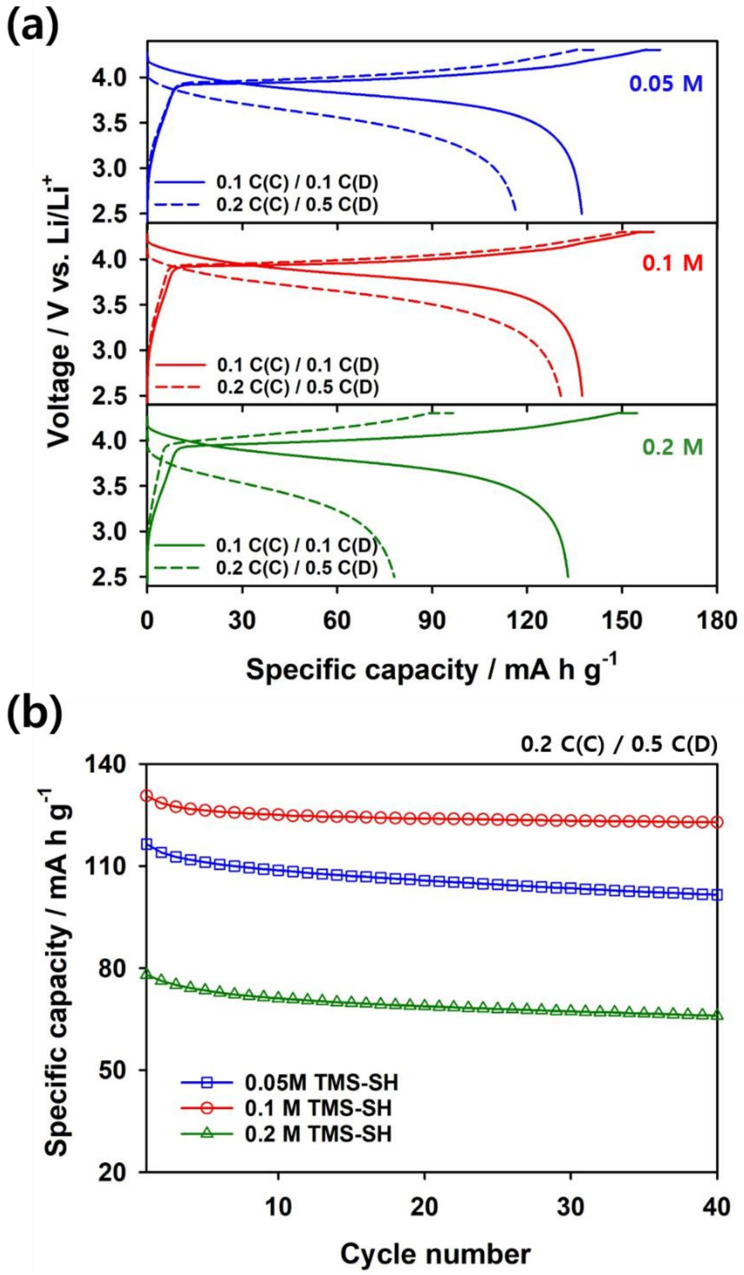

Figure S18. a) Voltage profiles of $LiCoO_2$ with TMS-LPSCl for various concentrations of TMS-SH, such as 0.05 M (blue line), 0.1 M (red line), and 0.2 M (green line), in the voltage range of 2.5 – 4.3 V (vs. Li/Li^+) at 30 °C. The cells were examined at the two current protocols for charge and discharge: (i) at a 0.1C rate (0.11 mA cm^{-2}, solid line) for charge and discharge, and (ii) at a 0.2 C rate (0.22 mA cm^{-2}) for charge and a 0.5 C rate (0.54 mA cm^{-2}) for discharge (dashed line). b) Cycle performance of $LiCoO_2$ with TMS-LPSCl for various concentrations of TMS-SH, such as 0.05 M (blue square), 0.1 M (red circle), and 0.2 M (green triangle), in the voltage range of 2.5 – 4.3 V (vs. Li/Li^+) at 30 °C. The cells were charged at a 0.2 C rate and discharged at a 0.5 C rate.

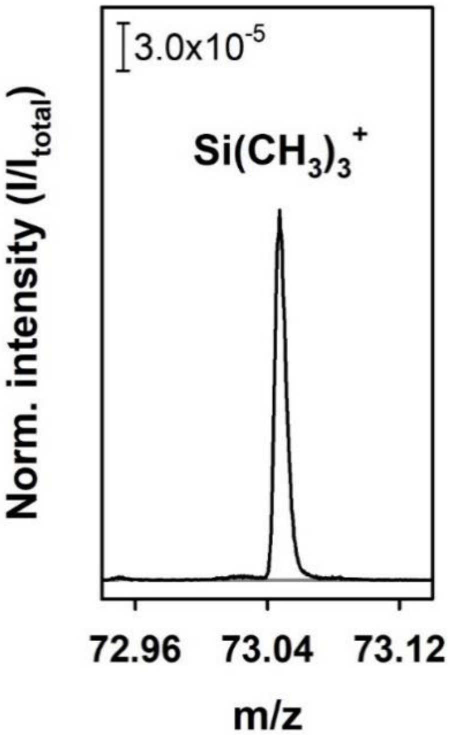

Figure S19. Normalized TOF-SIMS spectra of bare LPSCl and TMS-LPSCl for $Si(CH_3)_3^+$ fragments. Black and grey spectra represent TMS-LPSCl and bare LPSCl, respectively. The scale bars of normalized intensity are inserted in the figures.

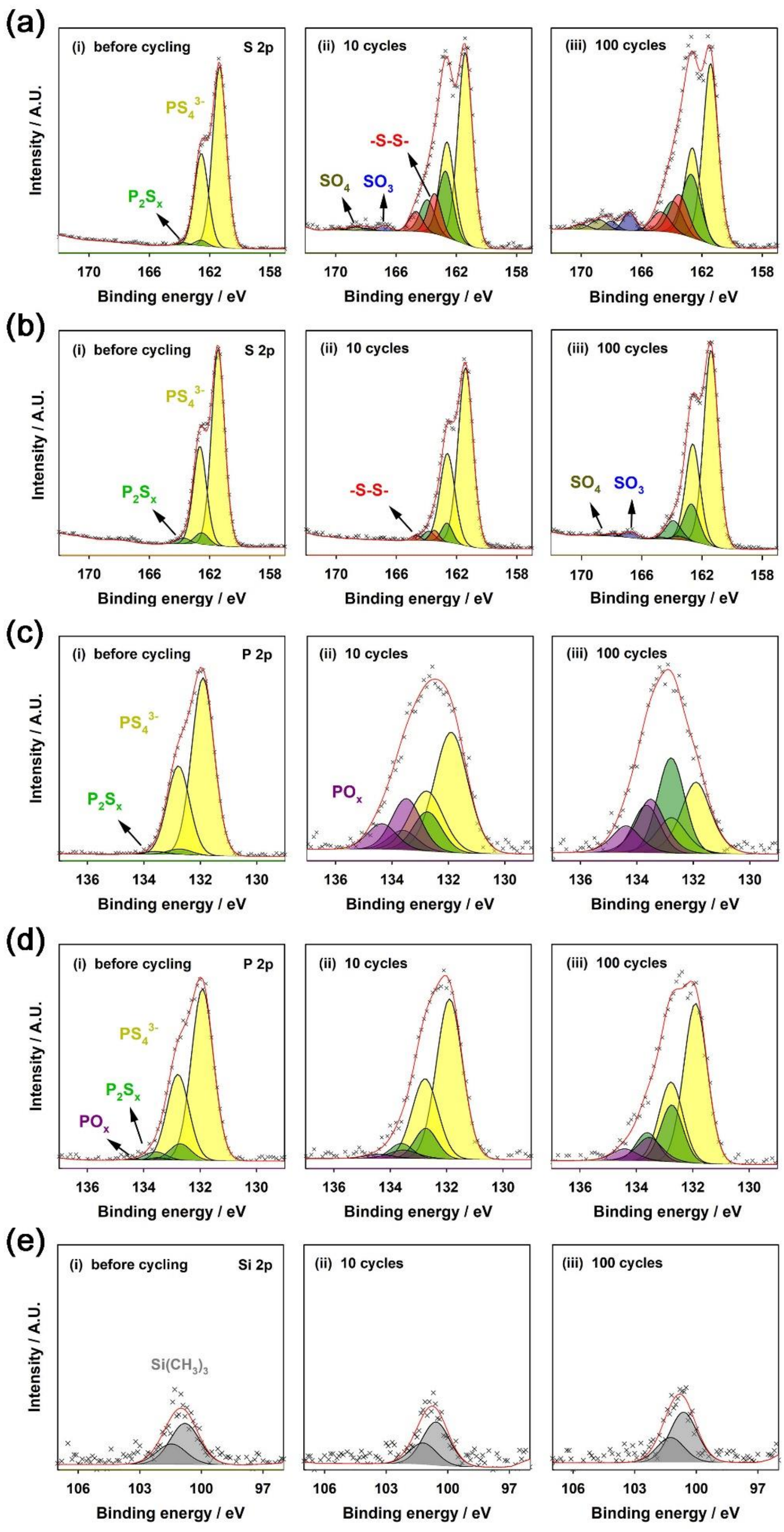

Figure S20. a-e) Ex situ XPS spectra of S 2p, P 2p, and Si 2p for bare LPSCl (a, c) and TMS-LPSCl (b, d, e) before cycling (i) and after 10 (ii) and 100 (iii) cycles.

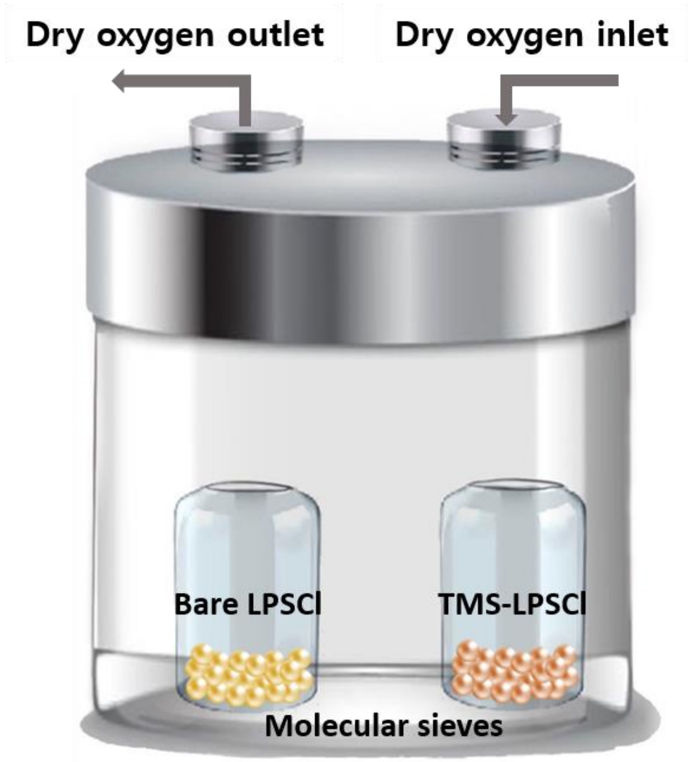

Figure S21. Schematic illustration for the home-made reactor to observe the chemical degradation of LPSCl powders in a dry oxygen atmosphere at room temperature.

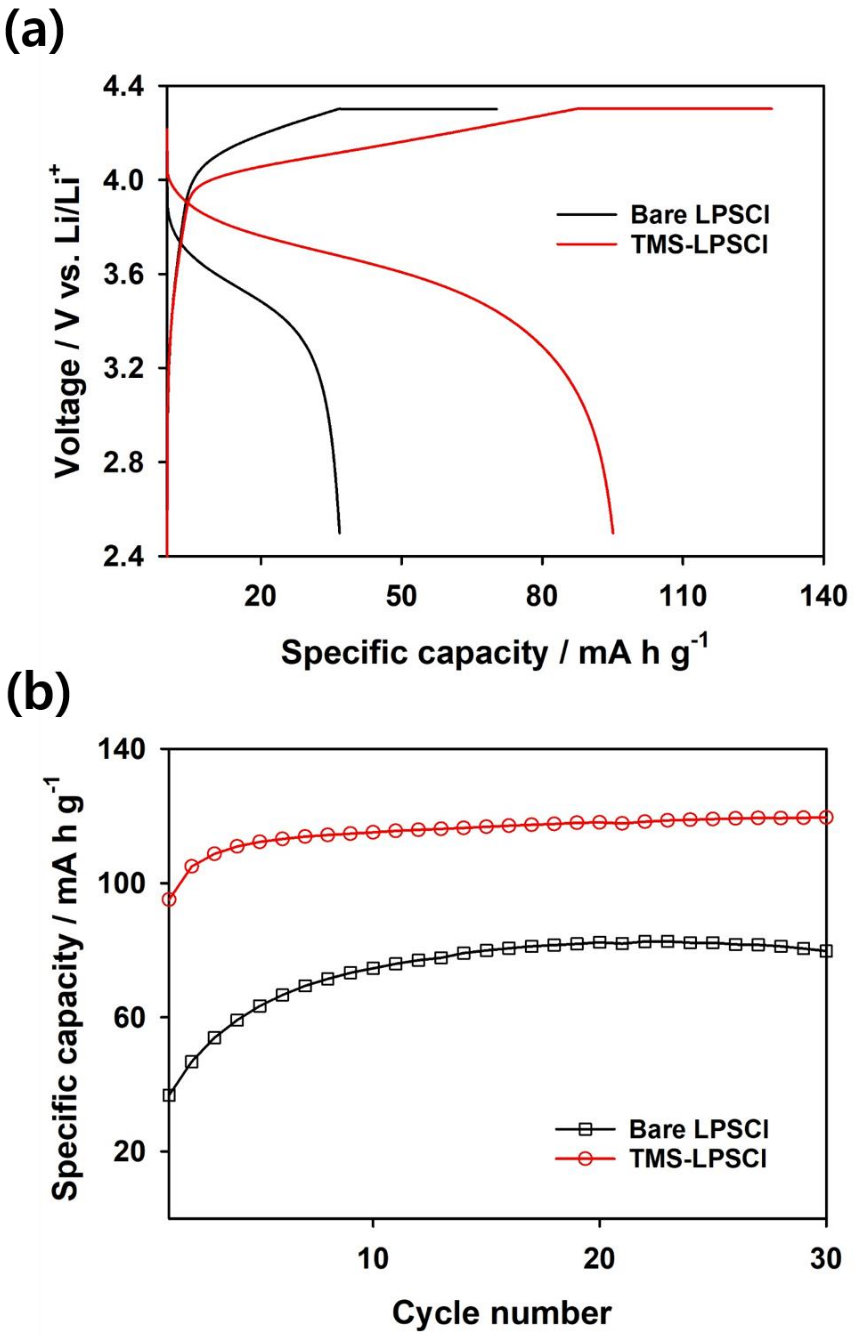

Figure S22. a) Voltage profiles and b) cycle performance of bare LPSCl and TMS-LPSCl, which were retrieved after storage in oxygen, at a current density of 0.1 C (0.11 mA cm^{-2}). The cells were examined in the voltage range of 2.5 – 4.3 V (vs. Li/Li^{+}) at 30 °C. Both powders were stored in a home-made container under a dry oxygen atmosphere for six days at room temperature.

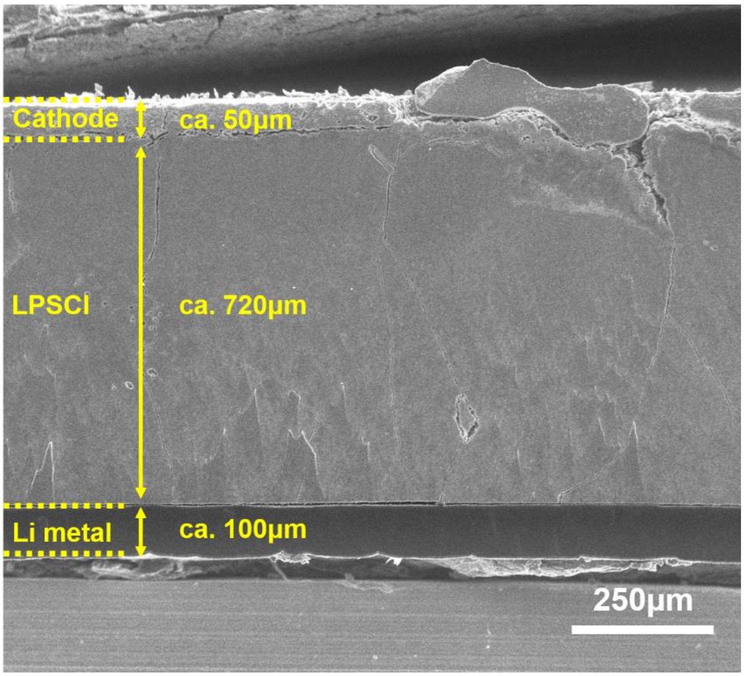

Figure S23. Cross-sectional SEM image of the Li | LPSCl | $LiCoO_2$ cell.

Table S1. Quantitative molar ratio of Li, P, and Si elements for LPSCl powders adsorbed with TMS-SH. The corresponding weight ratio of LPSCl and TMS-SH in LPSCl powders adsorbed with TMS-SH was calculated from the molar ratio of Li, P, and Si. Elemental analysis was performed using ICP-AES.

Atom	Li	P	Si
Molar ratio	5.974	1	0.094
Molecule	Li_6PS_5Cl		TMS-SH
Weight ratio	1		0.05

Table S2. The ionic and electronic conductivities of bare LPSCl and LPSCl adsorbed with TMS-SH and TMS-OH at room temperature.

	Electronic conductivity (mS cm^{-1})	Ionic conductivity (mS cm^{-1})
Bare LPSCl	8.22×10^{-6}	1.23
LPSCl adsorbed with TMS-SH	9.19×10^{-6}	0.62
LPSCl adsorbed with TMS-OH	1.98×10^{-5}	0.24

Table S3. Fitting parameters for the EIS data shown in Figure S5a.

	Bare LPSCl	LPSCl adsorbed with TMS-OH	LPSCl adsorbed with TMS-SH
R (ohm)	42.86	210.90	73.82
Standard deviation	0.17	0.23	0.15
goodness-of-fit ($\frac{\chi^2}{\lvert Z\rvert}$)	0.0096	0.0351	0.0046

Table S4. Fitting parameters for the EIS data shown in Figure S14.

	Bare LPSCl	THF-LPSCl	TMS-LPSCl
R (ohm)	42.86	66.10	73.82
Standard deviation	0.17	0.13	0.15
goodness-of-fit ($\frac{\chi^2}{\lvert Z\rvert}$)	0.0096	0.0715	0.0046

Table S5. Comparison of the electrochemical performances and cell parameters of $LiCoO_2$ cathode materials with sulfide-based solid electrolytes reported in recent literatures.

Cathode coating	Cathode loading / mg cm^{-2}	Electrolyte	Electrolyte thickness / µm (mg cm^{-2})	Anode type	Current density / mA cm^{-2} (C-rate)	Cycle number	Cycle retention / %	Temp. [°C]	Ref.
None	6.8	Li_6PS_5Cl	720 (113 mg cm^{-2})	Li	0.11 for charge, 1.1 for discharge	2000	85	30	This work
					0.44 for charge, 1.1 for discharge	200	95		
$LiNbO_3$	N/A	Li_6PS_5Cl	1200	Li	0.35	100	80.3	25	[S1]
None	4	$Li_{6.25}PS_4O_{1.25}Cl_{0.75}$	(75 mg cm^{-2})	Li	0.05	100	69.4	60	[S2]
					0.1	500	60		
$LiZrO_x$	4.5	Li_6PS_5Cl modified by PEGDME[a)]	800	Li	0.5	2600	~50[b)]	N/A	[S3]
None	N/A	$75Li_2S\cdot24P_2S_5\cdot1P_2O_5$	1200	Li	0.1 C	30	83.2	25	[S4]
$LiNbO_3$	3.6	$Li_7P_3S_{11}$	(153 mg cm^{-2})	Li	0.1	100	72.8	25	[S5]
				LiF coated Li			81.4		
$LiNbO_3$	7.5	Li_6PS_5Cl	1000	Li	0.1 C	100	47.0	N/A	[S6]
				Li_2Se			76.0		
$LiNbO_3$	2.7	LT-$Li_7P_2S_8I$[c)]	(191 mg cm^{-2})	Li	0.03	100	93	Room Temp.	[S7]
$LiNbO_3$	8.92	Li_3PS_4-Li_xSiS_y[d]	(127 mg cm^{-2})	Li	0.13	100	87.3	Room Temp.	[S8]
$LiNbO_3$	8.92	Li_6PS_5Cl-$Li_6PS_5Cl_{0.3}F_{0.7}$[d)]	(102 mg cm^{-2})	Li	0.13	50	95	Room Temp.	[S9]
$LiZrO_3$	1.74	Li_6PS_5Cl	(89 mg cm^{-2})	LiPFG[e)] coated Li	0.1 C	80	90	60	[S10]
$LiNbO_3$	N/A	$Li_{5.4}PS_{4.4}Cl_{1.2}Br_{0.4}$	N/A	Li	0.05	100	93	Room Temp.	[S11]
N/A	0.9	$Li_{10}GeP_2S_{12}$ with PVDF[f)] binder	30	Li	0.14	100	100	50	[S12]
N/A	2.7	Li_6PS_5Cl	(127 mg cm^{-2})	LiN_3 coated Li	0.1C	100	86.5	Room Temp.	[S13]
Li_2WO_4	8.9	Li_6PS_5Cl	1000	Li-In	0.14	100	93	25	[S14]
None							60		
None	9.8	Li_6PS_5Cl	(153 mg cm^{-2})	Ag coated Li	0.16	150	83.9	25	[S15]
$LiNbO_3$	8.9	$Li_{10}GeP_2S_{12}$	(166 mg cm^{-2})	Li-In	0.76	2000	78	35	[S16]
None	8.9	$Li_6PS_{4.75}ClO_{0.25}$	(166 mg cm^{-2})	Li-In	0.43	250	86	25	[S17]
None	18	Li_6PS_5Cl	(76 mg cm^{-2})	MP-Al-H[g)]	1.53	300	95.8	Room Temp.	[S18]
	19.6				6.7		77.2	50	
$Li_xZr_2(PO_4)_3$	8.9	Li_6PS_5Cl	(102 mg cm^{-2})	Li-In	0.13	100	95.5	30	[S19]

$Li_{1.175}Nb_{0.645}Ti_{0.4}O_3$	6.4	Li_6PS_5Cl	N/A	Li-In	0.45	1000	88.6	Room Temp.	[S20]
$LiNbO_3$	5.5	$Li_{10}GeP_2S_{12}$	(127 mg cm^{-2})	LiH_2PO_4 coated Li	0.1 C	550	86.7	25	[S21]
$Li_4Ti_5O_{12}$	36.9	$Li_{9.54}Si_{1.74}P_{1.44}S_{11.7}Cl_{0.3}$	(89 mg cm^{-2})	Li-In	3.14	200	88	50	[S22]
$LiNbO_3$	7 mg	$Li_{10}GeP_2S_{12}$	N/A	Li-In	0.13	100	71.8	Room Temp.	[S23]
$Li_2CoTi_3O_8$	7.64	$Li_{10}GeP_2S_{12}$	(102 mg cm^{-2})	Li-In	0.11	200	82.9	Room Temp.	[S24]
$Li_2TiO_3/Li_xB_yO_z$	8.91	$Li_{10}GeP_2S_{12}$	(102 mg cm^{-2})	Li-In	0.27	300	82.3	30	[S25]
N/A	10.2 – 12.7	CO_2-treated Li_6PS_5Cl	(102 mg cm^{-2})	Li-In	0.5 C	2100	89.4	Room Temp.	[S26]

a) a poly(ethylene glycol) dimethyl ether; b) an estimated value because it is not specified in the paper; c) a low temperature phase $Li_7P_2S_8I$ glass-ceramic electrolyte; d) a bi-layer separator; e) a robust artificial protective layer consisting of organic matrix embedded with inorganic Li_3N and LiF; f) a poly(vinylidene fluoride); g) a mechanically pre-lithiated aluminum foil.

Table S6. Fitting parameters for the EIS data shown in Figure S17b.

	TMS-SH 0.05M	TMS-SH 0.1M	TMS-SH 0.2M
R (ohm)	69.20	73.82	125.30
Standard deviation	0.22	0.15	0.17
goodness-of-fit ($\frac{\chi^2}{\lvert Z \rvert}$)	0.0035	0.0046	0.0591

Table S7. Quantitative molar ratio of Li, P, and Si elements for LPSCl powders adsorbed with TMS-SH for various concentrations of TMS-SH in THF (0.05 M and 0.2 M). The corresponding weight ratio of LPSCl and TMS-SH in LPSCl powders adsorbed with TMS-SH was calculated from the molar ratio of Li, P, and Si. Elemental analysis was performed using ICP-AES.

Concentration of TMS-SH (0.05 M)	Li	P	Si
Molar ratio	5.12	1	0.03
Molecule	Li_6PS_5Cl		TMS-SH
Weight ratio	1		0.02

Concentration of TMS-SH (0.2 M)	Li	P	Si
Molar ratio	5.26	1	0.21

Molecule	Li_6PS_5Cl	TMS-SH
Weight ratio	1	0.11

Table S8. Fitting parameters for the EIS data shown in Figure 9b and c

	Bare LPSCl				TMS-LPSCl			
	0 day	2 day	4 day	6 day	0 day	2 day	4 day	6 day
R (ohm)	42.86	95.61	101.90	113.40	73.82	95.20	97.39	99.53
deviation	0.172	0.163	0.165	0.156	0.148	0.142	0.152	0.140
goodness-of-fit ($\frac{\chi^2}{\|Z\|}$)	0.0096	0.0028	0.0089	0.0890	0.0046	0.0045	0.0343	0.0211

References:

[S1] G. Liu, W. Weng, Z. Zhang, L. Wu, J. Yang, X. Yao, *Nano Lett.* **2020**, 20, 6660.

[S2] H. Xu, G. Cao, Y. Shen, Y. Yu, J. Hu, Z. Wang, G. Shao, *Energy Environ. Mater.* **2020** 5, 852.

[S3] X. Yang, X. Gao, M. Jiang, J. Luo, J. Yan, J. Fu, H. Duan, S. Zhao, Y. Tang, R. Yang, *Angew. Chem.* **2022**, e202215680

[S4] Y. Tao, S. Chen, D. Liu, G. Peng, X. Yao, X. Xu, *J. Electrochem. Soc.* **2016**, 163, A96.

[S5] R. Xu, F. Han, X. Ji, X. Fan, J. Tu, C. Wang, *Nano Energy* **2018**, 53, 958.

[S6] H. Park, J. Kim, D. Lee, J. Park, S. Jo, J. Kim, T. Song, U. Paik, *Adv. Sci.* **2021**, 8, 2004204.

[S7] L. Wu, G. Liu, H. Wan, W. Weng, X. Yao, *J. Power Sources* **2021**, 491, 229565.

[S8] J. Liang, X. Li, Y. Zhao, L. V. Goncharova, W. Li, K. R. Adair, M. N. Banis, Y. Hu, T.-K. Sham, H. Huang, L. Zhang, S. Zhao, S. Lu, R. Li, X. Sun, *Adv. Energy Mater.* **2019**, 9, 1902125.

[S9] F. Zhao, Q. Sun, C. Yu, S. Zhang, K. Adair, S. Wang, Y. Liu, Y. Zhao, J. Liang, C. Wang, X. Li, X. Li, W. Xia, R. Li, H. Huang, L. Zhang, S. Zhao, S. Lu, X. Sun, *ACS Energy Lett.* **2020**, 5, 1035.

[S10] C. Wang, X. Sun, L. Yang, D. Song, Y. Wu, T. Ohsaka, F. Matsumoto, J. Wu, *Adv. Mater. Interfaces* **2021**, 8, 2001698.

[S11] Z. Zhang, Y. Tian, G. Liu, M. Wu, H. He, X. Yao, *J. Electrochem. Soc.* **2022**, 169, 040553.

[S12] G. Yu, Y. Wang, K. Li, D. Chen, L. Qin, H. Xu, J. Chen, W. Zhang, P. Zhang, Z. Sun, *Sustain. Energy Fuels* **2021**, 5, 1211.

[S13] Y. Liu, H. Su, M. Li, J. Xiang, X. Wu, Y. Zhong, X. Wang, X. Xia, C. Gu, J. Tu, *J. Mater. Chem. A* **2021**, 9, 13531.

[S14] Z. Sun, Y. Lai, N. lv, Y. Hu, B. Li, S. Jing, L. Jiang, M. Jia, J. Li, S. Chen, F. Liu, *Adv. Mater. Interfaces* **2021**, 8, 2100624.

[S15] B. Li, Z. Sun, N. Lv, Y. Hu, L. Jiang, Z. Zhang, F. Liu, *ACS Appl. Mater. Interfaces* **2022**, 14, 37738.

[S16] G. Lu, X. Li, Z. Wang, D. Song, H. Zhang, C. Li, L. Zhang, L. Zhu, *J. Power Sources* **2020**, 468, 228372.

[S17] Z. Sun, Y. Lai, N. lv, Y. Hu, B. Li, L. Jiang, J. Wang, S. Yin, K. Li, F. Liu, *ACS Appl. Mater. Interfaces* **2021**, 13, 54924.

[S18] Z. Fan, B. Ding, Z. Li, B. Hu, C. Xu, C. Xu, H. Dou, X. Zhang, *Small* **2022**, 18, 2204037.

[S19] L. Wang, X. Sun, J. Ma, B. Chen, C. Li, J. Li, L. Chang, X. Yu, T.-S. Chan, Z. Hu, M. Noked, G. Cui, *Adv. Energy Mater.* **2021**, 11, 2100881.

[S20] W. He, N. Ahmad, S. Sun, X. Zhang, L. Ran, R. Shao, X. Wang, W. Yang, *Adv. Energy Mater.* **2022**, n/a, 2203703.

[S21] Z. Zhang, S. Chen, J. Yang, J. Wang, L. Yao, X. Yao, P. Cui, X. Xu, *ACS Appl. Mater. Interfaces* **2018**, 10, 2556.

[S22] Z. Li, Z. Wang, Y. Miao, Y. Ma, H. Zhang, X. Shi, D. Song, L. Zhang, L. Zhu, *J. Power Sources* **2022**, 541, 231703.

[S23] C. Wang, X. Li, Y. Zhao, M. N. Banis, J. Liang, X. Li, Y. Sun, K. R. Adair, Q. Sun, Y. Liu, F. Zhao, S. Deng, X. Lin, R. Li, Y. Hu, T.-K. Sham, H. Huang, L. Zhang, R. Yang, S. Lu, X. Sun, *Small Methods* **2019**, 3, 1900261.

[S24] C.-W. Wang, F.-C. Ren, Y. Zhou, P.-F. Yan, X.-D. Zhou, S.-J. Zhang, W. Liu, W.-D. Zhang, M.-H. Zou, L.-Y. Zeng, X.-Y. Yao, L. Huang, J.-T. Li, S.-G. Sun, *Energy Environ. Sci.* **2021**, 14, 437.

[S25] L. Feng, Z.-W. Yin, C.-W. Wang, Z. Li, S.-J. Zhang, P.-F. Zhang, Y.-P. Deng, F. Pan, B. Zhang, Z. Lin, *Adv. Funct. Mater.* **2023**, n/a, 2210744.

[S26] X. Zhang, X. Li, S. Weng, S. Wu, Q. Liu, M. Cao, Y. Li, Z. Wang, L. Zhu, R. Xiao, D. Su, X. Yu, H. Li, L. Chen, Z. Wang, X. Wang, *Energy Environ. Sci.* **2023**, 16, 1091.
